# Multiomics analyses reveal *DARS1-AS1*/YBX1–controlled posttranscriptional circuits promoting glioblastoma tumorigenesis/radioresistance

**DOI:** 10.1126/sciadv.adf3984

**Published:** 2023-08-04

**Authors:** Caishang Zheng, Yanjun Wei, Qiang Zhang, Ming Sun, Yunfei Wang, Jiakai Hou, Peng Zhang, Xiangdong Lv, Dan Su, Yujie Jiang, Joy Gumin, Nidhi Sahni, Baoli Hu, Wenyi Wang, Xi Chen, Daniel J. McGrail, Chaolin Zhang, Suyun Huang, Han Xu, Junjie Chen, Frederick F. Lang, Jian Hu, Yiwen Chen

**Affiliations:** ^1^ Department of Bioinformatics and Computational Biology, The University of Texas MD Anderson Cancer Center, Houston, TX 77030, USA.; ^2^ Department of Cancer Biology, The University of Texas MD Anderson Cancer Center, Houston, TX 77030, USA.; ^3^ Department of Molecular and Cellular Biology, Baylor College of Medicine, Houston, TX 77030, USA.; ^4^ Lester and Sue Smith Breast Center, Baylor College of Medicine, Houston, TX 77030, USA.; ^5^ Dan L. Duncan Comprehensive Cancer Center, Baylor College of Medicine, Houston, TX 77030, USA.; ^6^ Department of Experimental Radiation Oncology, The University of Texas MD Anderson Cancer Center, Houston, TX 77030, USA.; ^7^ Department of Statistics, Rice University, Houston, TX 77005, USA.; ^8^ Department of Neurosurgery, The University of Texas MD Anderson Cancer Center, Houston, TX 77030, USA.; ^9^ Department of Epigenetics and Molecular Carcinogenesis, The University of Texas MD Anderson Cancer Center, Houston, TX 77030, USA.; ^10^ Program in Quantitative and Computational Biosciences (QCB), Baylor College of Medicine, Houston, TX 77030, USA.; ^11^ Department of Neurological Surgery, University of Pittsburgh School of Medicine, Pittsburgh, PA 15213, USA.; ^12^ Pediatric Neurosurgery, UPMC Children's Hospital of Pittsburgh, Pittsburgh, PA 15224, USA.; ^13^ Molecular and Cellular Cancer Biology Program, UPMC Hillman Cancer Center, Pittsburgh, PA 15232, USA.; ^14^ Department of Biostatistics, The University of Texas MD Anderson Cancer Center, Houston, TX 77030, USA.; ^15^ Center for Immunotherapy and Precision Immuno-Oncology, Cleveland Clinic, Cleveland, OH 44195, USA.; ^16^ Lerner Research Institute, Cleveland, OH 44195, USA.; ^17^ Department of Systems Biology, Department of Biochemistry and Molecular Biophysics, and Center for Motor Neuron Biology and Disease, Columbia University, New York, NY 10032, USA.; ^18^ Department of Human and Molecular Genetics, Institute of Molecular Medicine, VCU Massey Cancer Center, Virginia Commonwealth University, School of Medicine, Richmond, VA 23298, USA.; ^19^ Quantitative Sciences Program, MD Anderson Cancer Center UTHealth Graduate School of Biomedical Sciences, Houston, TX 77030, USA.; ^20^ The Center for Cancer Epigenetics, The University of Texas MD Anderson Cancer Center, Houston, TX 77030, USA.; ^21^ Cancer Biology Program, MD Anderson Cancer Center UTHealth Graduate School of Biomedical Sciences, Houston, TX 77030, USA.; ^22^ Neuroscience Program, MD Anderson Cancer Center UTHealth Graduate School of Biomedical Sciences, Houston, TX 77030, USA.

## Abstract

The glioblastoma (GBM) stem cell–like cells (GSCs) are critical for tumorigenesis/therapeutic resistance of GBM. Mounting evidence supports tumor-promoting function of long noncoding RNAs (lncRNAs), but their role in GSCs remains poorly understood. By combining CRISPRi screen with orthogonal multiomics approaches, we identified a lncRNA *DARS1-AS1*–controlled posttranscriptional circuitry that promoted the malignant properties of GBM cells/GSCs. Depleting *DARS1-AS1* inhibited the proliferation of GBM cells/GSCs and self-renewal of GSCs, prolonging survival in orthotopic GBM models. *DARS1-AS1* depletion also impaired the homologous recombination (HR)–mediated double-strand break (DSB) repair and enhanced the radiosensitivity of GBM cells/GSCs. Mechanistically, *DARS1-AS1* interacted with YBX1 to promote target mRNA binding and stabilization, forming a mixed transcriptional/posttranscriptional feed-forward loop to up-regulate expression of the key regulators of G_1_-S transition, including E2F1 and CCND1. *DARS1-AS1*/YBX1 also stabilized the mRNA of *FOXM1*, a master transcription factor regulating GSC self-renewal and DSB repair. Our findings suggest *DARS1-AS1*/YBX1 axis as a potential therapeutic target for sensitizing GBM to radiation/HR deficiency–targeted therapy.

## Teaser

Multiomics analyses reveal lncRNA/RBP-mediated post-transcriptional circuits promoting glioblastoma tumorigenesis/radioresistance.

## INTRODUCTION

Glioblastoma (GBM), a high-grade glioma (grade IV), is the most prevalent and malignant primary brain tumor in adults. Upon diagnosis, the standard treatment of GBM includes maximal surgical resection, followed by radiation therapy administered concurrently with temozolomide. The therapeutic benefit from the standard treatment remains limited, and the median survival of GBM patients is around 15 months. GBM has characteristics of enhanced cell proliferation and high propensity of invasion/diffuse infiltration (*1*). It is also featured by aberrant activation of DNA damage response/repair (DDR) pathway (*2*, *3*) and resistance to chemotherapy/radiation therapy (*1*). The aberrant activation of DDR pathway in GBM enables tumor cells to cope with both exogenous ionizing radiation and endogenous replication stress (*2*). Substantial evidence has revealed a subpopulation of highly tumorigenic GBM cells, the glioblastoma stem cell–like cells (GSCs) (*4*), that have unique functional characteristics including the capability of self-renewal and differentiation into other cell types, persistent proliferation, and tumor initiation upon secondary transplantation (*4*). In addition to its critical role in tumor initiation, the GSCs confer therapeutic resistance of GBM in response to chemotherapy and radiation therapy (*3*, *4*). Previous studies revealed important role of transcriptional regulatory circuits in governing the functional characteristics of GSCs (*5*). In contrast, the posttranscriptional regulatory circuits critical for tumorigenesis and chemo-/radio-resistance of GSCs and how they are integrated with the transcription circuits remain largely unknown.

There are more than 15,000 long (>200 nucleotides) noncoding RNA (lncRNA) genes in the human genome. They are an emerging class of regulatory RNAs, some of which can mediate tumor-promoting/tumor-suppressing effects and serve as independent diagnostic/prognostic biomarkers in cancer (*6*). In addition, lncRNAs play important roles in cell-fate decisions, such as controlling the pluripotency and differentiation of embryonic stem cells (*7*, *8*). Different from microRNAs (miRNAs), lncRNAs regulate gene expression through diverse mechanisms. The most well-established mechanism of lncRNA regulation is epigenetic regulation (*9*). Mounting evidence supports that lncRNAs in the cytoplasm can regulate the stability (*10*)/translation (*11*) of mRNAs, whereas our understanding of the lncRNA/RNA binding protein (RBP) complex–mediated posttranscriptional regulation remains limited compared with that of the epigenetic regulation.

Past studies from us (*12*) and others (*13*) revealed that many lncRNAs showed dysregulated expression and/or harbored somatic genetic alternations in GBM, suggesting a potential role of lncRNAs in the molecular pathogenesis of GBM. However, the role and function mechanism of the lncRNA/RBP-mediated regulatory circuits in determining the functional characteristics of GSCs underlying GBM pathogenesis remains poorly understood. To fill this gap, we combined CRISPR (clustered regularly interspaced short palindromic repeat) interference (CRISPRi) (*14*, *15*) with large-scale computational analysis of The Cancer Genome Atlas (TCGA) (*16*) to systematically identify the functional lncRNAs with clinically relevant expression in GBM. We further integrated multiomics approaches with molecular and functional assays to identify a posttranscriptional regulatory circuitry controlled by one of the top lncRNA hits from our CRISPRi screen and its associated RBP, which critically regulates the proliferation, tumorigenesis, and radioresistance of GSCs/GBM cells.

## RESULTS

### Integrating transcriptome analysis and CRISPRi screen to identify functional lncRNAs in GBM

**Fig. 1.**
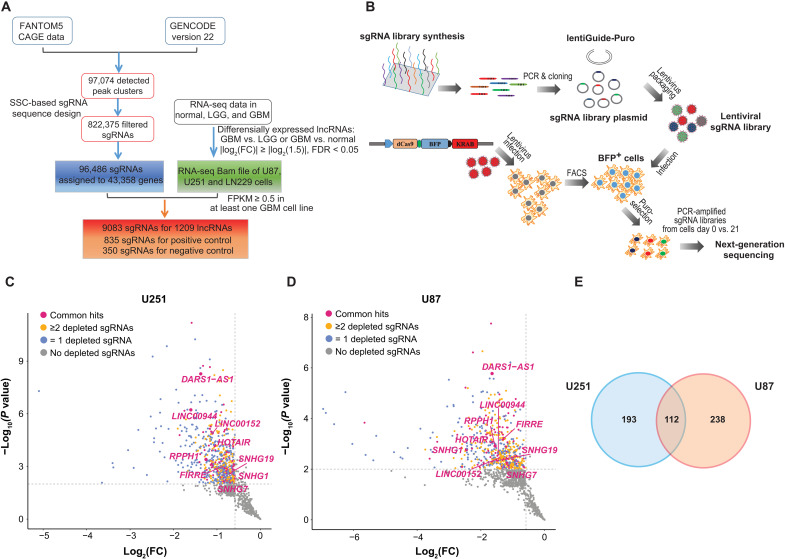
An integrative strategy for identifying functional lncRNAs in GBM with clinically relevant expression. (**A**) Workflow diagram depicting the integrative strategy for identifying GBM-associated lncRNAs and designing CRISPRi sgRNA library. (**B**) Schema of the workflow for construction of lentiviral vectors encoding sgRNA library and experimental design of CRISPRi screens. The scatterplot showing the statistical significance −log_10_ (*P* value) and log_2_ (fold change) [log_2_(FC)] between D21 and D0, for the representative negatively selected sgRNA of the corresponding lncRNA genes in (**C**) U87 and (**D**) U251 cells. The lncRNA genes with zero, one, and at least two significantly depleted sgRNAs are colored in gray, blue, and yellow in the indicated cell lines, respectively. The lncRNA genes with at least two significantly depleted sgRNAs in both cell lines are shown in red. (**E**) Venn diagram showing the overlap of the lncRNA genes with at least two significantly negatively selected targeting sgRNAs in U251 and U87.

To systematically identify the GBM dependency on lncRNAs with clinically relevant expression, we performed CRISPRi screens on lncRNAs that showed differential expression between GBM tumors and low-grade glioma (LGG) tumors or normal brain tissues (Materials and Methods, Fig. 1A). The CRISPRi system used in our screens relies on the catalytically inactive Cas9 (dCas9) protein fused with a KRAB repressor domain (dCas9-KRAB), targeted through single-guide RNAs (sgRNAs), to the specific loci within the promoter-proximal regions to repress lncRNA transcription (*14*). We first integrated the FANTOM5 cap analysis of gene expression (CAGE) data with GENCODE V22 transcriptome annotation to define the 5′ ends or transcription starting sites (TSSs) on a transcriptome-wide level as previously described (*17*) and then designed the sgRNAs targeting TSS-proximal regions using the Sequence Scan for CRISPR (SSC) method (Materials and Methods) (*18*). A total of 97,074 CAGE clusters were assigned as the TSSs of transcripts in GENCODE V22. After filtering out sgRNA sequences of low quality, 96,486 uniquely mapped sgRNAs that target the TSS-proximal regions of 43,358 genes were selected. We further performed differential gene expression analysis to identify the lncRNA genes with deregulated expression in GBM tumors compared with LGG tumors or normal brain tissues [|log_2_fold change| ≥ log_2_(1.5), false discovery rate (FDR) < 0.05], based on RNA-sequencing (RNA-seq) data (fig. S1, A and B; Materials and Methods). Among the identified dysregulated lncRNA genes, we further filtered out the ones with low expression in GBM cell lines U87, U251, and LN229 [fragments per kilobase of transcript per million mapped reads (FPKM) ≤ 0.5 in all three cell lines]. Last, we selected 9083 sgRNAs targeting 1209 lncRNA genes as well as the positive and negative control sgRNAs for CRISPRi sgRNA library construction (Fig. 1A and table S1).

The CRISPRi screens were conducted in two GBM cell lines U251 and U87 that stably express the dCas9-KRAB fusion protein, in a similar way to our previous study (Fig. 1B) (*19*). Briefly, the cells transduced with the lentiviral vectors encoding the sgRNA library were selected with puromycin (puro). The puro-selected cells were passaged for 21 days. The abundance change of individual sgRNAs between the cells collected on day 0 (D0) and day 21 (D21) was quantified by next-generation sequencing (Materials and Methods). The sequencing libraries including the one from the library plasmid exhibited low Gini indexes (fig. S1, C and D), suggesting a general uniformity of the sgRNA coverage. As expected for the working positive controls, we observed a notable depletion in the abundance of the sgRNAs targeting positive control core essential genes in final (D21) cell populations compared with the initial (D0) ones, but no difference in sgRNA abundance between negative control and positive control sgRNAs/lncRNA-targeting sgRNAs in the libraries collected from D0 or library plasmid (fig. S1, E to G). The sgRNA abundance from the three replicates showed a significant correlation (Pearson correlation coefficient *r >* 0.75, *P* < 2.2 × 10^−16^) with each other on D0 and D21 (fig. S1H).

We used MAGeCK (*20*) to assess the statistical significance of the level of sgRNA depletion (Materials and Methods). There were 305 lncRNA genes in U251 cells and 350 lncRNA genes in U87 cells that had at least two significantly negatively selected targeting sgRNAs [log_2_(fold change) ≤ −log_2_(1.5), *P* < 0.01, FDR < 0.05], with 112 of them being common ones (Fig. 1, C to E, and table S1). The common lncRNA genes included the ones with well-established function in development and/or disease, such as *HOTAIR* (*21*) and *FIRRE* (*22*). The common functional lncRNA genes also included multiple small nucleolar RNA host gene (*SNHG*) family members, such as *SNHG1*, *SNHG7*, and *SNHG19*. Among the shared 112 lncRNA genes, we further selected those showing a significantly elevated expression in GBM compared with normal brain tissues [log_2_(fold change) ≥ log_2_(1.5), FDR < 0.01] based on RNA-seq data (Materials and Methods), resulting in a total of 57 lncRNA gene hits that represent the clinically relevant candidates for GBM dependency.

### Validating the top-ranked screen hits overexpressed in GBM

**Fig. 2.**
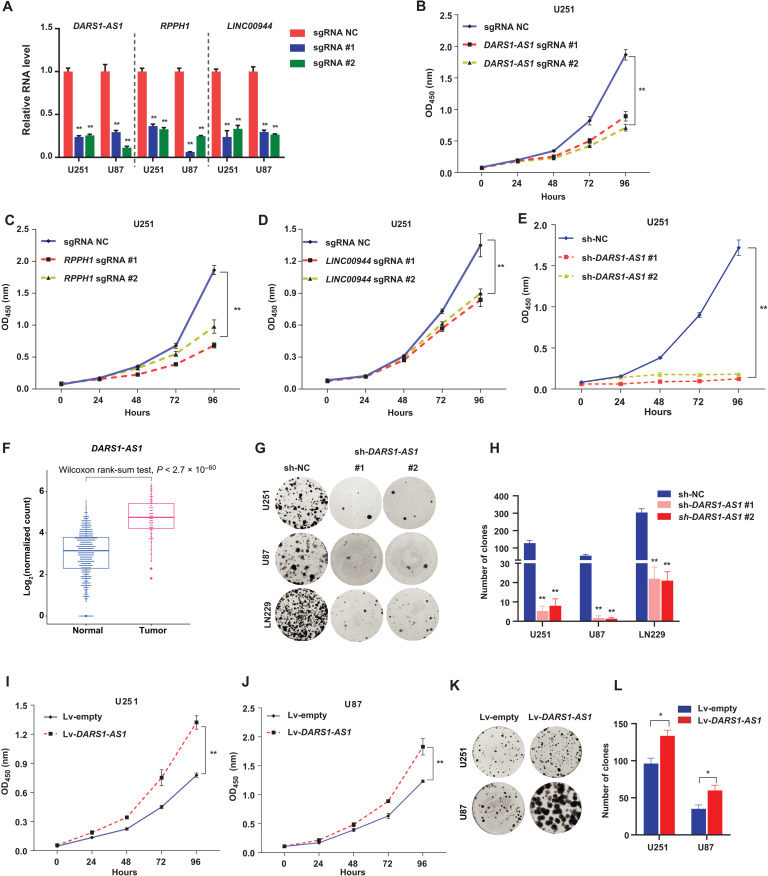
Validation of the top lncRNA hits in GBM cells. (**A**) RT-qPCR analysis of knockdown efficiency for the indicated sgRNAs targeting *DARS1-AS1*, *RPPH1*, and *LINC00944* compared with the negative control sgRNA (sg-NC) in U251 and U87 with stable expression of dCas9-KRAB fusion protein, where GAPDH was used as an internal control. The growth of U251 cells with stable expression of dCas9-KRAB transduced with sg-NC/sgRNAs targeting (**B**) *DARS1-AS1*, (**C**) *RPPH1*, or (**D**) *LINC00944* was monitored (OD_450_ absorbance for WST-8 formazan) every 24 hours with CCK-8 assay for 96 hours. (**E**) Growth of the U251 cells transduced with the negative control shRNA (sh-NC)/*DARS1-AS1*–targeting shRNAs was measured with CCK-8 assay for the indicated time intervals. (**F**) Boxplots showing *DARS1-AS1* expression in GBM tumors and normal brain tissues based on TCGA and GTEx RNA-seq data. The statistical significance of difference was assessed by Wilcoxon rank sum test. (**G**) Representative pictures of clonogenic growth and (**H**) the bar graph quantifying the colonies formed by U251, U87, and LN229 cells transduced with sh-NC or shRNAs targeting *DARS1-AS1*, after cells were cultured for 2 weeks. The growth of (**I**) U251 and (**J**) U87 cells transduced with an empty lentiviral lincXpress vector (Materials and Methods) control (Lv-empty) or *DARS1-AS1* overexpression lincXpress vector (Lv-*DARS1-AS1*) was monitored every 24 hours with CCK-8 assay for 96 hours. (**K**) Representative pictures of clonogenic growth and (**L**) the bar graph quantifying the colonies formed by U251 or U87 cells transduced with Lv-empty or Lv-*DARS1-AS1*, after cells were cultured for 2 weeks. Data in (A) to (E) and (H) are shown as mean ± SD (*n* = 3). **P < 0.01 by one-way ANOVA with Dunnett’s multiple comparison test. Data in (I), (J), and (L) are shown as mean ± SD (*n* = 3). **P < 0.01 or *P < 0.05 by Student’s *t* test.

To validate the CRISPRi screen results, we selected three lncRNA genes *DARS1-AS1*, *LINC00944*, and *RPPH1* that were among the top-ranked ones out of the 57 total hits (table S1) and do not yet have an established function in GBM for functional validation. For each lncRNA gene, we selected the top two sgRNAs that showed the strongest growth inhibitory effect in CRISPRi screens (Materials and Methods and table S2). The effective depletion of these lncRNAs by either of the two gene-specific sgRNAs (Fig. 2A) inhibited the growth of U251 and U87 cells with stable expression of dCas9-KRAB fusion protein (Fig. 2, B to D, and fig. S2, A to C). To control for the possibility that the observed CRISPRi-mediated loss-of-function phenotype for these lncRNA genes may be caused by CRISPRi-mediated off-target effect (*23*), we used RNA interference (RNAi), an alternative approach that acts on RNAs for independent validation. We found that small interfering RNA (siRNA)/short hairpin RNA (shRNA)–mediated knockdown of these lncRNA genes also inhibited the growth of GBM cells (Fig. 2E and fig. S2, D to L).

We next focused on *DARS1-AS1* (also named *DARS-AS1*), an antisense lncRNA gene for further functional and mechanistic characterization. To confirm the overexpression of *DARS1-AS1* in GBM tumors compared with normal brain tissues, we analyzed the RNA-seq data of the normal brain tissues from the Genotype-Tissue Expression (GTEx) (*24*) project, a different dataset from our initial differential lncRNA expression analysis. *DARS1-AS1* showed a consistent up-regulation in the GBM tumors compared with the normal brain tissues from GTEx (Fig. 2F). We also found that the expression level of *DARS1-AS1* was higher in the mesenchymal subtype tumors than in the classical and proneural subtype tumors in GBM (*25*), and its higher expression was significantly associated with shorter overall survival of GBM patients (log-rank test, *P* = 0.0023; fig. S3, A and B). This association remained significant (*P* < 0.015) when additional covariates of sex, age, and GBM subtype were included in the multivariate Cox proportional hazards regression analysis (Materials and Methods). To confirm that *DARS1-AS1* is a noncoding RNA gene, we used two computational methods CPAT (*26*) and PLEK (*27*) to assess the coding probability of its transcript sequences. All *DARS1-AS1* transcripts were predicted to be noncoding by both methods. Consistent with its loss-of-function phenotype in cell growth assay, RNAi-mediated knockdown of *DARS1-AS1* impaired the clonogenic capacity of GBM cells (Fig. 2, G and H). As an antisense lncRNA gene can often act in cis to regulate the expression of its neighboring gene, we assessed the effect of RNAi-mediated inhibition of *DARS1-AS1* expression on the RNA level of its neighboring gene *DARS1*. We observed that siRNA-mediated *DARS1-AS1* knockdown did not affect the expression of *DARS1* (fig. S2M), suggesting that *DARS1-AS1* may act in trans. We further performed 5′ and 3′ RACE (rapid amplification of 5′/3′ complementary DNA ends; fig. S2N) and confirmed that the experimentally determined 5′ and 3′ ends of *DARS1-AS1* transcript (NR_110199.1) were consistent with its original RefSeq annotation. With the confirmed full-length transcript, we investigated *DARS1-AS1* gain-of-function phenotype in cell growth and clonogenic assay by stably overexpressing its full-length transcript in GBM cells. Consistent with the loss-of-function data, *DARS1-AS1* RNA overexpression (fig. S2O) promoted the growth and colony formation (Fig. 2, I to L) of GBM cells, further supporting a growth-promoting function of *DARS1-AS1*.

### *DARS1-AS1* is required for the growth/self-renewal of patient-derived GSCs, and its inhibition prolongs survival in orthotopic tumor models

**Fig. 3.**
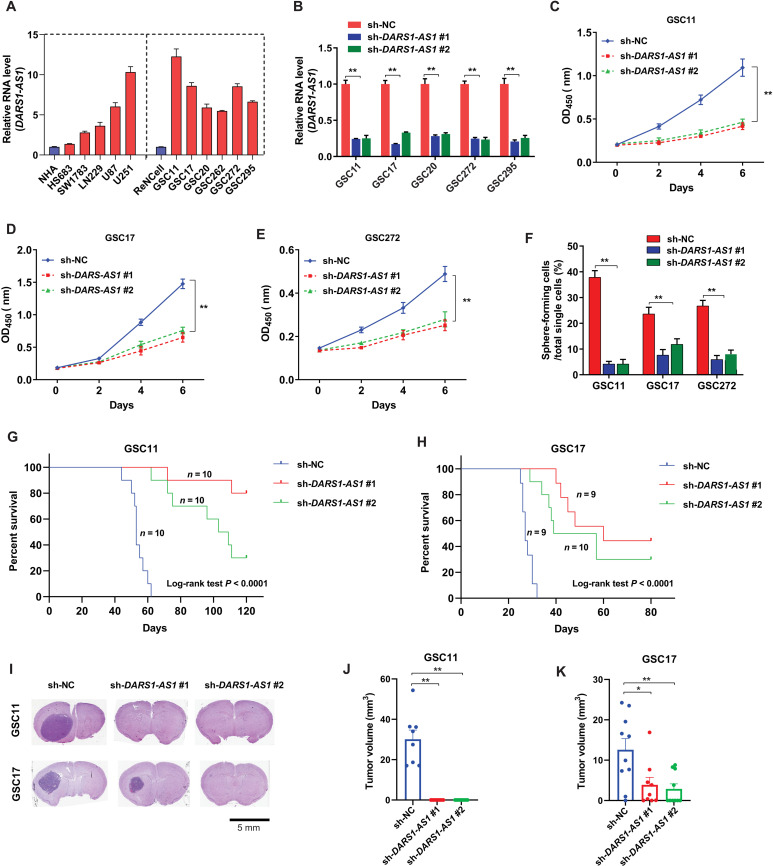
*DARS1-AS1* promotes growth/self-renewal of patient-derived GSCs and orthotopic tumor formation. (**A**) RT-qPCR analysis of RNA-level *DARS1-AS1* expression in the immortalized NHAs, the LGG cell lines (Hs683 and SW1783), the immortalized human neural progenitor cell line (ReNCell), GBM cells (U87, U251, and LN229), and GSCs (GSC11, GSC17, GSC20, GSC272, and GSC295). (**B**) The knockdown efficiency of the indicated *DARS1-AS1*–targeting shRNAs compared with the negative control shRNA (sh-NC) was determined by RT-qPCR in GSC11, GSC17, GSC20, GSC272, and GSC295 cells. The growth of (**C**) GSC11, (**D**) GSC17, and (**E**) GSC272 cells transduced by the negative control shRNA (sh-NC) or individual *DARS1-AS1*–targeting shRNAs was measured by CCK-8 assays every 2 days for 6 days. (**F**) Percentages of GSC cells transduced with sh-NC or *DARS1-AS1*–targeting shRNAs that can form neurospheres from single cells determined by self-renewal assay. The Kaplan-Meier survival curves of the mice (*P* < 0.0001, log-rank test) with intracranial injection of patient-derived (**G**) GSC11 and (**H**) GSC17 cells stably expressing sh-NC (10 GSC11 mice, 9 GSC17 mice, blue), sh-*DARS1-AS1* #1 (10 GSC11 mice, 9 GSC17 mice, red), and sh-*DARS1-AS1* #2 (10 GSC11 mice, 10 GSC17 mice, green). (**I**) Thirty (or 21) days after GSC11 (or GSC17) cells stably expressing sh-NC or sh-*DARS1-AS1* #1/sh-*DARS1-AS1*#2 were intracranially grafted into athymic nude mice, the mouse brains were harvested, fixed, embedded, and stained by H&E. Representative images of H&E-stained tumor section are shown. Scale bar, 5 mm. Tumor volumes were calculated as indicated in Materials and Methods for (**J**) GSC11 and (**K**) GSC17 cells expressing sh-NC (8 GSC11 mice, 10 GSC17 mice, blue), sh-*DARS1-AS1* #1 (10 GSC11 mice, 9 GSC17 mice, red) or sh-*DARS1-AS1* #2 (10 GSC11 mice, 10 GSC17 mice, green). Data are shown as mean ± SD. **P < 0.01 or *P < 0.05 by one-way ANOVA with Dunnett’s multiple comparison test. Data in (B) to (F) are shown as mean ± SD (*n* = 3). **P < 0.01 by one-way ANOVA with Dunnett’s multiple comparison test.

In accordance with its up-regulated expression of GBM tumors compared with normal brain tissues, *DARS1-AS1* showed a higher expression in GBM cell lines (U251, U87, and LN229) and patient-derived GSCs (GSC11, GSC17, GSC20, GSC262, GSC272, and GSC295), in comparison with the LGG cell lines (Hs683 and SW1783), the immortalized normal human astrocytes (NHAs), and ReNcell, a neural stem cell line (Fig. 3A).

We next investigated the function of *DARS1-AS1* in patient-derived GSCs. Consistent with the results for GBM cells, we found that shRNA-mediated depletion of *DARS1-AS1* (Fig. 3B) inhibited the GSC growth (Fig. 3, C to E). In addition, using a self-renewal assay as previously described (*28*) (Materials and Methods), we found that shRNA-mediated depletion of *DARS1-AS1* in GSCs impaired their self-renewal capability, indicated by a significantly reduced percentage of tumor-sphere formation from single cells (Fig. 3F). Moreover, the survival of the nude mice that were intracranially grafted with the GSCs transduced with *DARS1-AS1*–targeting shRNAs was significantly prolonged compared with the mice grafted with the GSCs transduced with scrambled negative control shRNAs (Fig. 3, G and H; log-rank test, *P* < 0.0001). Consistently, shRNA-mediated depletion of *DARS1-AS1* in GSCs inhibited GSC-derived orthotopic tumor formation in vivo (Fig. 3, I to K). Collectively, these results demonstrate that *DARS1-AS1* critically regulates the growth and self-renewal of patient-derived GSCs in vitro and GSC-derived orthotopic tumor formation in vivo.

### *DARS1-AS1* interacts with YBX1, and this interaction is important for mediating *DARS1-AS1* function

**Fig. 4.**
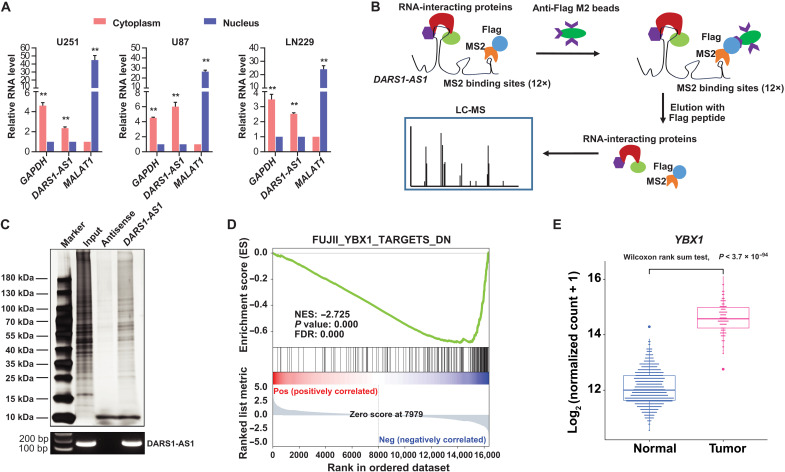
Integrative analysis of AP-MS and RNA-seq data identifies YBX1 as a *DARS1-AS1*–associated protein with overlapping downstream targets. (**A**) The RNA level of *DARS1-AS1* in the nuclear and cytoplasmic fraction of U251 cells was measured by RT-qPCR. *MALAT1* RNA and *GAPDH* mRNA were used as positive controls for nuclear and cytoplasmic fraction, respectively. (**B**) Schematic diagram showing the workflow identifying *DARS1-AS1*–associated proteins with MS2bs-tagged RNA affinity purification, coupled with MS analysis. The *DARS1-AS1*/protein complex was immunoprecipitated with anti-FLAG antibody in the formaldehyde–cross-linked GBM cells stably expressing MS2bs-tagged *DARS1-AS1* and FLAG-tagged MS2 proteins, followed by the MS analysis of the eluted proteins. (**C**) The proteins were retrieved by the pull-down of MS2bs-tagged *DARS1-AS1* RNA, and the negative control antisense RNA was visualized by silver staining and subjected to MS analysis. The RNAs retrieved by the RNA pull-down experiments were detected with semiquantitative RT-PCR. (**D**) GSEA analysis of the RNA-seq data generated with siRNA-mediated *DARS1-AS1* knockdown revealed enrichment of a YBX1–down-regulated gene signature. NES, normalized enrichment score. (**E**) Boxplots showing the expression of *YBX1* in GBM tumors and normal brain tissues based on TCGA and GTEx RNA-seq data. The statistical significance of the expression difference between GBM tumors and normal brain tissues was assessed by Wilcoxon rank sum test.

To explore the potential molecular mechanism underlying the tumor-promoting function of *DARS1-AS1*, we first determined the subcellular localization of *DARS1-AS1* RNAs in GBM cells. The reverse transcription quantitative polymerase chain reaction (RT-qPCR) assay in different subcellular fractions showed that *DARS1-AS1* RNAs were more enriched in the cytoplasm than in the nucleus (Fig. 4A), suggesting a potential cytoplasmic function of *DARS1-AS1*. To systematically identify *DARS1-AS1*–interacting proteins, we performed affinity purification (AP) using an anti-FLAG antibody followed by mass spectrometry (MS) in formaldehyde–cross-linked GBM cells expressing FLAG-tagged bacteriophage MS2 coat protein and MS2 binding site (MS2bs)–tagged sense/antisense sequence of *DARS1-AS1* transcript NR_110199.1 (Fig. 4B, Materials and Methods) (*10*). The cross-linking with formaldehyde that covalently secures protein-protein and protein-RNA interactions before the cells are lysed enables native cellular context to be preserved. Silver staining showed an enrichment of specific bands from the AP of MS2bs-tagged *DARS1-AS1* RNAs in comparison with that of the negative control MS2bs-tagged *DARS1-AS1* antisense sequence (Fig. 4C). In addition, the *DARS1-AS1* RNAs can be specifically recovered from the AP of MS2bs-tagged *DARS1-AS1* RNAs (Fig. 4C). We identified a total of 362 proteins with at least two unique peptides identified by MS in the *DARS1-AS1* RNA pull-down, but no detected unique peptides in the pull-down of antisense *DARS1-AS1* control (table S3).

In parallel with the AP-MS experiments, we generated RNA-seq data to identify the protein-coding genes up- or down-regulated by siRNA-mediated *DARS1-AS1* knockdown in GBM cells (Materials and Methods). We performed gene set enrichment analysis (GSEA) (*29*) using the RNA-seq data to identify the gene signatures/sets that were enriched in the differentially expressed genes caused by *DARS1-AS1* knockdown (table S4). GSEA analysis revealed that target genes down-regulated by YBX1 (also known as YB1 or YB-1), an RBP (*30*) that was identified from our AP-MS experiments, were significantly enriched in the down-regulated genes by *DARS1-AS1* knockdown in our GSEA analysis (Fig. 4D). In addition, this YBX1 gene signature showed the best normalized enrichment score among all gene sets/signatures that were related to the *DARS1-AS1*–associated proteins identified in the AP-MS experiments (table S4). This finding suggests that YBX1 may be a candidate *DARS1-AS1*–interacting protein that co-regulates the RNA expression of their common downstream targets. YBX1 has been suggested to play diverse roles in posttranscriptional regulation, including regulating splicing (*31*, *32*), RNA stability (*33*, *34*), and translation (*35*, *36*), and has been shown to be involved in complex diseases such as cancer (*34*, *35*) and neurological disease [e.g., Rett syndrome (*37*)]. Recent studies have revealed an important role of YBX1 in promoting the tumor growth and drug resistance in GBM (*38*, *39*). Consistently, we found that YBX1 was up-regulated in GBM tumors compared with normal brain tissues (Fig. 4E and fig. S4A). In addition, knockdown of *YBX1* (fig. S4B) inhibited the growth and colony formation of GBM cells (fig. S4, C to F) as well as the growth and self-renewal of GSCs (fig. S4, G to I).

**Fig. 5.**
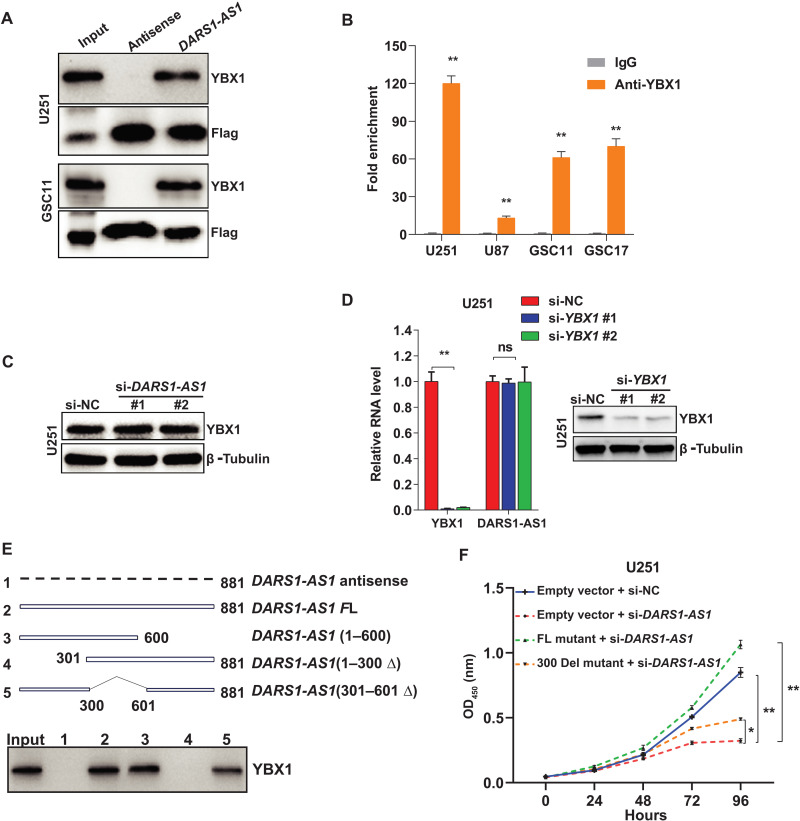
Validation and characterization of the interaction between *DARS1-AS1* and YBX1. (**A**) RNA pull-down coupled with Western blot validated the interaction between *DARS1-AS1* and YBX1 that was identified from MS analysis. (**B**) RIP with an anti-YBX1/anti-IgG antibody followed by RT-qPCR validated the association of YBX1 with *DARS1-AS1*, where anti-IgG antibody was used as a negative control. (**C**) The protein level of YBX1 was determined by Western blot in U251 cells transfected with the negative control siRNA (si-NC) or individual *DARS1-AS1*–targeting siRNAs, where β-tubulin was used as a loading control. (**D**) RT-qPCR analysis of *YBX1* and *DARS1-AS1* RNA level in U251 cells transfected with the negative control siRNA (si-NC) or individual *YBX1*-targeting siRNAs. Right: The YBX1 protein expression was determined by Western blot. (**E**) RNA pull-down of the MS2bs-tagged antisense, full-length, and serial deletion mutants of *DARS1-AS1* RNA followed by anti-YBX1 Western blotting. The three serial deletion mutants of *DARS1-AS1* RNA were generated by deleting 601 to 881, 1 to 300, or 301 to 600 bp, respectively. (**F**) U251 cells stably transduced with the vectors expressing full-length mutant *DARS1-AS1* resistant to siRNAs (FL mutant), the deletion mutant with a deletion of 1 to 300 bp (300 del) or the empty vector control, were transfected with the negative control siRNA (si-NC) or siRNAs targeting *DARS1-AS1* and were cultured for 4 days. The cell growth was monitored each day with CCK-8 assay. Data in (B) are shown as mean **±** SD (*n* = 3). **P < 0.01 by Student’s *t* test. Data in (D) and (F) are shown as mean**±** SD (*n* = 3). **P < 0.01, *P < 0.05, or ns, not significant (*P* > 0.05) by one-way ANOVA with Dunnett’s multiple comparison test.

We further validated the interaction between *DARS1-AS1* and YBX1 using *DARS1-AS1* RNA pull-down followed by anti-YBX1 Western blotting and YBX1 RNA immunoprecipitation (RIP) coupled with RT-qPCR (RIP–RT-qPCR) (Fig. 5, A and B, Materials and Methods). Depletion of *DARS1-AS1* did not affect *YBX1* expression at the RNA or protein level (Fig. 5C and fig. S4, J to L), and depletion of *YBX1* did not affect *DARS1-AS1* expression either (Fig. 5D). These results indicate that *DARS1-AS1* and YBX1 interact with but not regulate the expression of each other, suggesting that they may form a lncRNA/RBP complex. To identify the regions in the *DARS1-AS1* RNA that was important for its interaction with YBX1, we generated serial deletion mutants with the deletion of 1 to 300, 301 to 600, or 601 to 881 base pairs (bp), respectively. The pull-down of MS2bs-tagged full-length and different deletion mutants of *DARS1-AS1* RNA followed by anti-YBX1 Western blotting showed that deletion of 1 to 300 bp of *DARS1-AS1* RNA abolished its interaction with YBX1, indicating that this region was necessary for *DARS1-AS1*:YBX1 interaction (Fig. 5E). To evaluate the functional significance of the *DARS1-AS1*:YBX1 interaction, we performed rescue experiments for the cell growth defect caused by si-*DARS1-AS1* treatment, by overexpressing the full-length siRNA-resistant *DARS1-AS1* RNA (NR_1100199.1) or the mutant RNA with the deletion of 1 to 300 bp, respectively. Overexpressing the full-length siRNA-resistant RNA fully rescued the si-*DARS1-AS1*–mediated cell growth defect, whereas overexpressing the deletion (1 to 300 bp) mutant that showed a defective interaction with YBX1 was only able to partially rescue this growth defect, indicating that *DARS1-AS1*:YBX1 interaction is important for mediating the growth-promoting function of *DARS1-AS1* (Fig. 5F). To determine which domain of YBX1 interacts with *DARS1-AS1*, we constructed a series of expression vectors for green fluorescent protein (GFP) and GFP-tagged full-length or truncation mutant YBX1 (fig. S4M). The RNA pull-down assay using the U251 cells ectopically expressing GFP or GFP-tagged full-length/individual truncation mutant YBX1, followed by anti-GFP Western blot (Supplementary Methods), revealed that both the cold shock domain (CSD) and the C-terminal domain (CTD) of YBX1 interacted with *DARS1-AS1* RNA (fig. S4N). We were unable to determine whether the alanine/proline-rich domain of YBX1 interacted with *DARS1-AS1* because of technical difficulties in expressing this domain.

### *DARS1-AS1*/YBX1 regulates a common gene expression program controlling cell cycle progression

**Fig. 6.**
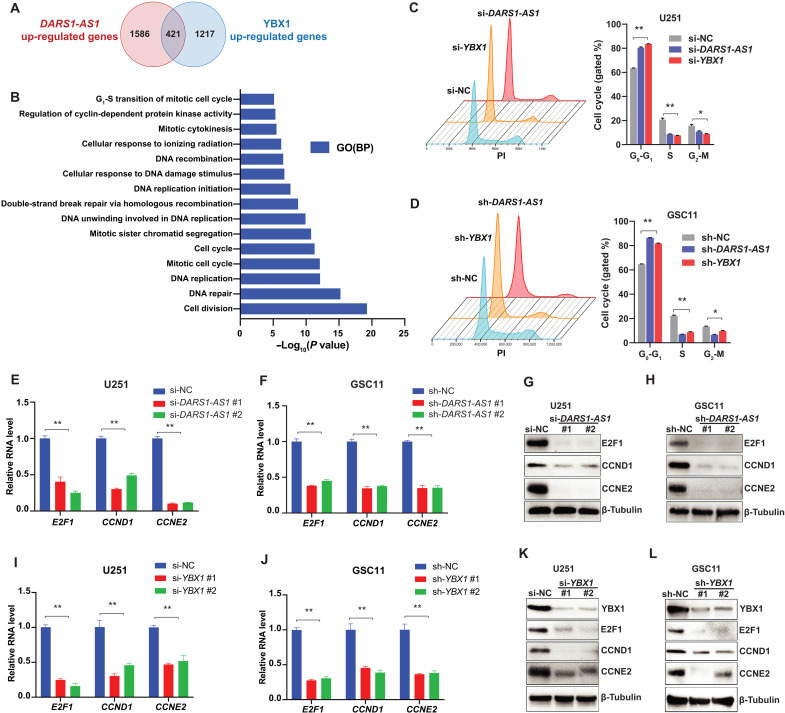
*DARS1-AS1*/YBX1 regulates a common gene expression program controlling cell cycle progression. (**A**) Venn diagram showing the overlap of the genes up-regulated by *DARS1-AS1* or YBX1 (i.e., down-regulated by siRNA-mediated depletion of *DARS1-AS1* or *YBX1*). (**B**) The bar plot showing the top enriched GO biological processes ranked by –log_10_(*P* value) of the GO enrichment analysis of the 421 genes down-regulated by the *DARS1-AS1*/YBX1 axis. Flow cytometry–based cell cycle analyses were performed for (**C**) U251 and (**D**) GSC11 cells that were transfected/transduced with the negative control siRNA(si-NC)/shRNA (sh-NC) or siRNA/shRNA targeting *DARS1-AS1* and were stained with PI. The percentages of the cells in the indicated cell cycle stages were quantified and shown in the bar graph. RT-qPCR analysis of RNA level of the established regulators of cell cycle, *E2F1*, *CCND1*, and *CCNE2* in (**E**) U251 and (**F**) GSC11 cells transfected/transduced with the negative control si-NC/sh-NC or siRNA/shRNA targeting *DARS1-AS1*. The protein level of *E2F1*, *CCND1*, and *CCNE2* was determined by Western blotting in (**G**) U251 and (**H**) GSC11 cells, with/without RNAi-mediated *DARS1-AS1* depletion. RT-qPCR analysis of the RNA level of *E2F1*, *CCND1*, and *CCNE2* in (**I**) U251 and (**J**) GSC11 cells transfected/transduced with the negative control si-NC/sh-NC or siRNA/shRNA targeting *YBX1*. The protein level of *E2F1*, *CCND1*, and *CCNE2* was determined by Western blotting in (**K**) U251 and (**L**) GSC11 cells, with/without RNAi-mediated *YBX1* depletion. Data in (C) to (F), (I), and (J) are shown as mean**±**SD (*n* = 3). **P < 0.01 or *P < 0.05 by one-way ANOVA with Dunnett’s multiple comparison test.

To identify the common downstream targets that are up-regulated by *DARS1-AS1*/YBX1 and may play a tumor-promoting role in GBM, we further performed RNA-seq experiments in the absence/presence of siRNA-mediated *YBX1* knockdown. We found 421 common protein-coding genes (table S4) that were down-regulated (Fig. 6A) by siRNA-mediated depletion of *YBX1* and *DARS1-AS1* [log_2_fold change ≤−log_2_(1.5), FDR < 0.05]. Consistent with our GSEA analysis, there was a statistically significant overlap (~25%; Fisher’s exact test, *P* < 2.2 × 10^−16^) between the genes down-regulated by siRNA-mediated *YBX1* and *DARS1-AS1* knockdown (Fig. 6A), supporting that *DARS1-AS1* and YBX1 co-regulated a significant fraction of downstream targets. The gene ontology (GO) enrichment analysis revealed that the common targets up-regulated by *DARS1-AS1*/YBX1 were enriched in the biological processes of DNA replication, cell cycle, and DNA repair, including double-strand break (DSB) repair via homologous recombination (HR; Fig. 6B and fig. S5A).

To determine the role of the *DARS1-AS1*/YBX1 axis in regulating cell cycle progression, we performed flow cytometry–based cell cycle analysis with propidium iodide (PI) DNA staining (Materials and Methods). We found that siRNA-mediated depletion of *DARS1-AS1* or *YBX1* increased the fractions of GBM cells (U251 and U87) or GSCs (GSC11 and GSC272) arrested at G_0_-G_1_ phase (Fig. 6, C and D, and fig. S5, B and C). In accordance with the cell cycle analysis, our RNA-seq data showed a down-regulation of multiple established regulators of G_1_-S transition upon siRNA-mediated depletion of *DARS1-AS1* (fig. S5A). We further confirmed that the siRNA-mediated depletion of *DARS1-AS1* or *YBX1* reduced the expression of *E2F1*, *CCND1*, and *CCNE2*, the key regulators of G_1_-S transition (*40*), at both the RNA and protein levels (Fig. 6, E to L, and fig. S5, D to K). Together, these data indicate that the *DARS1-AS1*/YBX1 axis promoted cell proliferation by up-regulating the expression of key regulators of G_1_-S transition.

### *DARS1-AS1*/YBX1 regulates the expression of the genes promoting HR-mediated DSB repair and modulates radiosensitivity

**Fig. 7.**
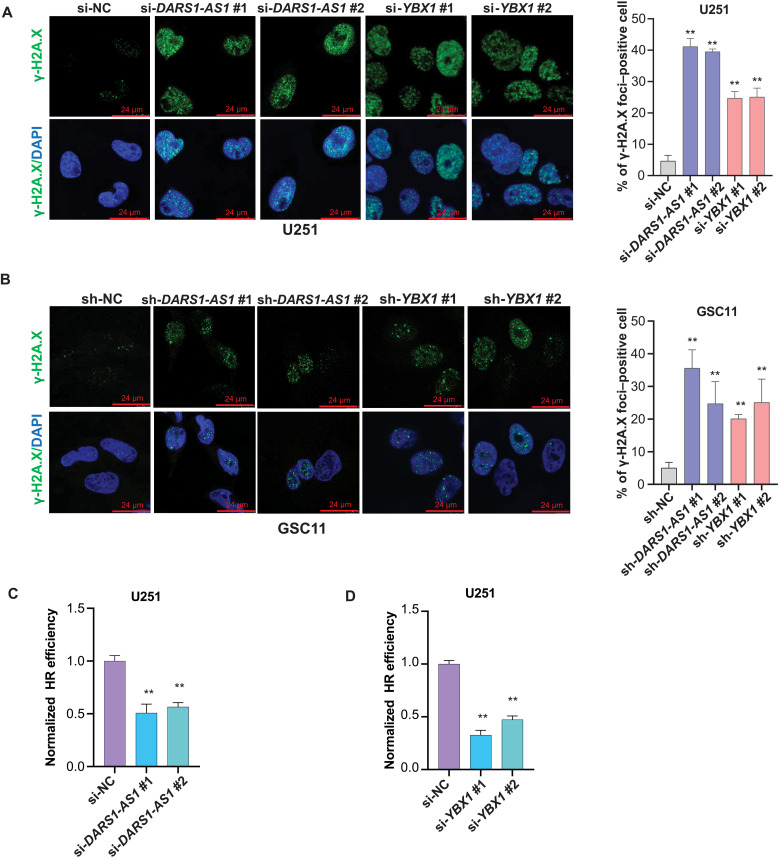
*DARS1-AS1*/YBX1 promotes HR-mediated DSB repair. The representative images of nuclear γ-H2AX foci detected by the immunofluorescence staining with anti–γ-H2AX (green) in the DAPI-stained nuclei (blue) from (**A**) U251 cells transfected with the negative control siRNA (si-NC) or the *DARS1-AS1*/*YBX1*–targeting siRNAs or from (**B**) GSC11 cells transduced with the negative control shRNA (sh-NC) or the *DARS1-AS1*/*YBX1*–targeting shRNAs. Scale bars, 24 μm. The percentages of γ-H2AX foci–positive cells (>5 foci in the nucleus) were shown as bar plots for the indicated conditions. The effect of siRNA-mediated knockdown of (**C**) *DARS1-AS1* or (**D**) *YBX1* compared with si-NC on the HR repair efficiency was assessed in U251 cells using a DR-GFP assay (Materials and Methods). Data in (A) to (D) are shown as mean ± SD (*n* = 3). **P < 0.01 by one-way ANOVA with Dunnett’s multiple comparison test.

**Fig. 8.**
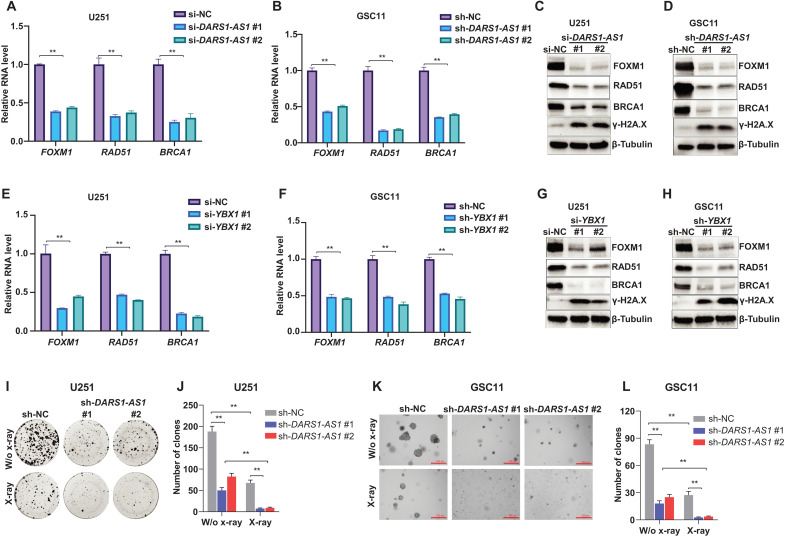
*DARS1-AS1*/YBX1 regulates expression of the genes promoting HR-mediated DSB repair and modulated radiosensitivity. RT-qPCR analysis of the RNA level of the known regulators for HR-mediated DSB repair *FOXM1*, *RAD51*, and *BRCA1* in (**A**) U251 and (**B**) GSC11 cells transfected/transduced with the negative control si-NC/sh-NC or individual siRNAs/shRNAs targeting *DARS1-AS1*. The protein level of *FOXM1*, *RAD51*, and *BRCA1* as well as the γ-H2AX level was determined by Western blotting in (**C**) U251 and (**D**) GSC11 cells with/without RNAi-mediated *DARS1-AS1* depletion. RT-qPCR analysis of the RNA level of HR-mediated DSB repair–related genes *FOXM1*, *RAD51*, and *BRCA1* in (**E**) U251 and (**F**) GSC11 cells transfected/transduced with the negative control si-NC/sh-NC or individual siRNAs/shRNAs targeting *YBX1*. The protein level of *FOXM1*, *RAD51*, and *BRCA1* as well as the γ-H2AX level were determined by Western blotting in (**G**) U251 and (**H**) GSC11 cells with/without RNAi-mediated *YBX1* depletion. (**I**) Representative pictures of clonogenic growth and (**J**) bar graph quantifying the colonies formed by U251 cells that were transduced with the sh-NC or shRNAs targeting *DARS1-AS1*. (**K**) Representative pictures of soft agar colony formation and (**L**) bar graph quantifying the colonies formed in soft agar by GSC11 cells that were transduced with the sh-NC or shRNAs targeting *DARS1-AS1*. Scale bar, 500 μm. Data in (A), (B), (E), (F), (J), and (L) are shown as mean **±**SD (*n* = 3). **P < 0.01 by one-way ANOVA with Dunnett’s multiple comparison test.

Aside from the regulators of cell cycle, our RNA-seq data revealed an enrichment of the regulators of HR-mediated DSB repair among the genes that were up-regulated by the *DARS1-AS1*/YBX1 axis (Fig. 6B and fig. S5A). Therefore, we hypothesize that the *DARS1-AS1*/YBX1 axis may promote the HR-mediated repair of DSBs caused by endogenous stress (e.g., replication stress) or exogenous ionizing radiation, and depleting *DARS1-AS1* or *YBX1* may lead to an increase of DSB level in GBM cells/GSCs. To test this hypothesis, we first performed immunofluorescence staining to assess the effect of depleting *DARS1-AS1* or *YBX1* on the formation of nuclear γ-H2AX (phosphorylation of the Ser^139^ residue of the histone variant H2AX) foci, a highly sensitive and specific marker of DSBs (Materials and Methods) (*41*). Consistent with our hypothesis, the siRNA-mediated knockdown of *DARS1-AS1* or *YBX1* greatly increased the number of γ-H2AX foci–positive cells (Fig. *7*, A and B). Next, we performed the direct-repeat (DR)–GFP HR reporter assay (*42*) (Materials and Methods) to evaluate the effect of depleting DARS1-AS1 or YBX1 on the HR repair of DSBs. We found that knockdown of *DARS1-AS1* or *YBX1* significantly reduced HR repair efficiency in U251 (Fig. *7*, C and D) and U2OS cells (fig. S6, A and B). Meanwhile, the knockdown of *DARS1-AS1*/*YBX1* increased the total γ-H2AX level and reduced the expression of *FOXM1*, *RAD51*, and *BRCA1*, the established regulators of HR-mediated DSB repair (*43*–*46*), at the RNA and protein level in GBM cells/GSCs (Fig. 8, A to H, and fig. S6, C to F). FOXM1 is not only a direct transcriptional activator of RAD51 (*46*), a key player in HR-mediated DSB repair, but also a master regulator of GSC self-renewal (*47*), suggesting an important role of FOXM1 in mediating *DARS1-AS1*/YBX1 regulation of both HR-mediated DSB repair and self-renewal in GSCs.

The aberrant activation of DNA damage response pathway (*2*, *3*) enables GBM cells/GSCs to cope with DSBs induced by endogenous stress (e.g., replication stress) or ionizing radiation and plays an important role in the therapeutic resistance of GBM to the chemotherapy/radiation therapy. With the observation that *DARS1-AS1* up-regulated the expression of several key regulators of HR-mediated DSB repair, we sought to determine the role of *DARS1-AS1* in modulating radiosensitivity of GBM cells/GSCs. We found that siRNA/shRNA-mediated suppression of *DARS1-AS1* expression led to a reduction in the number of colonies formed by GBM cells in clonogenic assay as well as a marked decrease in the number of colonies formed by GSCs in soft agar colony formation assay (Materials and Methods), following x-ray radiation treatment (Fig. 8, I to L, and fig. S6, G to J). These data indicated that inhibiting *DARS1-AS1* greatly increases the sensitivity of GBM cells/GSCs to ionizing radiation, which might be potentially exploited therapeutically to enhance the response of GBM to radiation therapy.

### *DARS1-AS1* promoted YBX1 binding to the key common targets to increase their mRNA stability

**Fig. 9.**
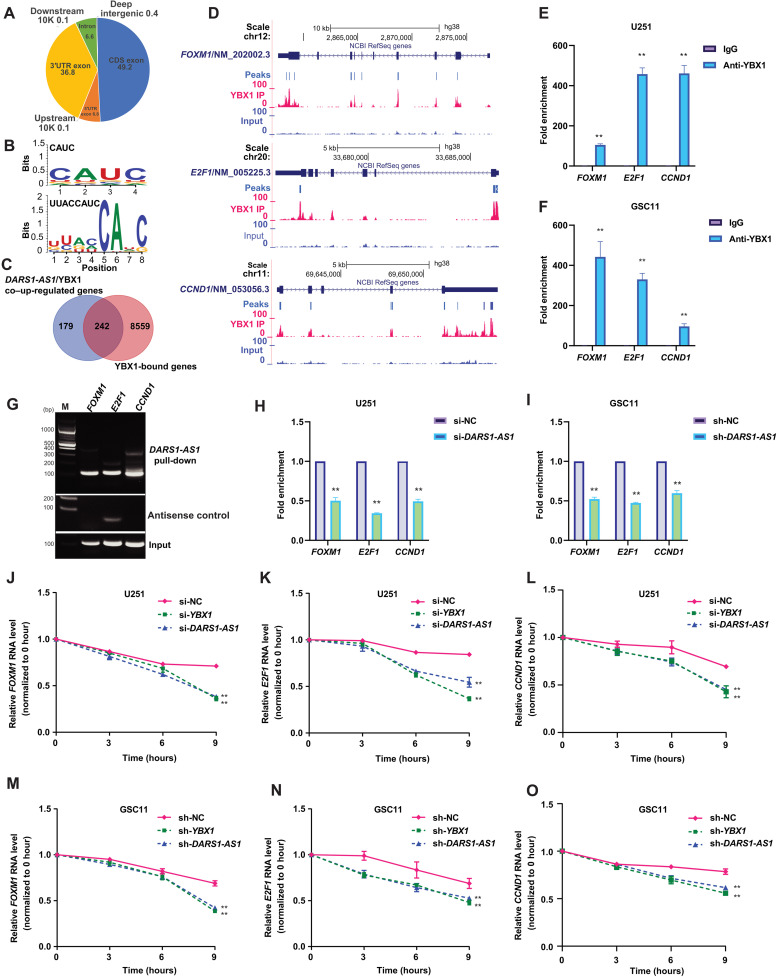
*DARS1-AS1* promotes YBX1 binding to the key common targets to increase their mRNA stability. (**A**) Genome-wide distribution of the YBX1 eCLIP-seq peaks over different elements. (**B**) The top-ranked 4- or 6-bp-long motif and the top-ranked motif of 8, 10, or 12 bp discovered de novo from the top 2000 YBX1 eCLIP-seq peaks by HOMER (v4.11-2). (**C**) Venn diagram showing the overlap between the 421 protein-coding genes down-regulated by siRNA-mediated knockdown of *DARS1-AS1* and *YBX1* and the genes whose mRNAs harbored at least one YBX1 eCLIP-seq peak. (**D**) The signal track of the YBX1 eCLIP-seq and the corresponding input were shown for the transcripts of *E2F1*, *CCND1*, and *FOXM1*. RIP with an anti-YBX1/anti-IgG antibody followed by RT-qPCR validated the association of YBX1 with *E2F1*, *CCND1*, and *FOXM1* mRNA in (**E**) U251 and (**F**) GSC11 cells, where anti-IgG antibody was used as a negative control. (**G**) The *E2F1*, *CCND1*, and *FOXM1* mRNA retrieved from the pull-down of MS2bs-tagged *DARS1-AS1* RNA, the negative control antisense RNA, or input in U251 cells was detected by semiquantitative RT-PCR. The fold enrichment of the YBX1 RIP signal normalized by the input on *E2F1*, *CCND1*, and *FOXM1* mRNA was determined in (**H**) U251 and (**I**) GSC11 cells with/without RNAi-mediated *DARS1-AS1* knockdown. After transcription inhibition with actinomycin D (0 hour), the relative RNA level (normalized by the level at 0 hour) of *FOXM1*, *E2F1*, and *CCND1* in U251 cells (**J** to **L**) transfected with si-NC/si-*YBX1*/si-*DARS1-AS1* or GSC11 cells (**M** to **O**) transduced with sh-NC/sh-*YBX1*/sh-*DARS1-AS1* at 0, 3, 6, and 9 hours was determined by RT-qPCR. Data in (E), (F), (H), and (I) are shown as mean **±**SD (*n* = 3). **P < 0.01 by Student’s *t* test. Data in (J) to (O) are shown as mean **±**SD (*n* = 3). **P < 0.01 by one-way ANOVA with Dunnett’s multiple comparison test.

To shed light on the mechanism whereby the *DARS1-AS1*/YBX1 axis regulates its downstream targets, we performed enhanced ultraviolet (UV) cross-linking and immunoprecipitation (CLIP) followed by sequencing (eCLIP-seq) (*48*) experiments (fig. S6K) with anti-FLAG antibody in the U251 cells stably expressing FLAG-tagged YBX1 (Materials and Methods). The eCLIP-seq data revealed a total of 42,947 transcriptome-wide YBX1 binding sites (table S5). The YBX1 binding sites were dominantly localized in the exonic regions, including coding exons (49.2%), 3′ untranslated regions (3′UTRs) (36.8%), and 5′UTRs (6.8%), with only a small fraction in introns (6.6%) (Fig. 9A). The motif enrichment analysis based on the top-ranked YBX1 binding sites revealed that the most enriched 4- or 6-bp-long motif is CAUC, which is the known core RNA binding motif of YBX1 (Fig. 9B) (*32*). Moreover, the top-ranked de novo motif of 8, 10, or 12 bp was UUACCAUC (Fig. 9B and table S5) and its top two best-matched known motifs were the YBX1 RNA binding motif identified with RNAcompete method (*30*). These results supported a good quality of our eCLIP-seq data. We found that 242 (~57%) of the 421 genes up-regulated by both *DARS1-AS1* and YBX1 were bound by YBX1 (Fig. 9C and table S5), suggesting that they may be direct targets of YBX1.

YBX1 plays a versatile role in regulating RNA splicing, stability, and translation. On the basis of our finding that the *DARS1-AS1*/YBX1 axis up-regulated the RNA-level expression of the critical regulators of cell cycle and HR-mediated DSB repair, we hypothesize that the *DARS1-AS1*/YBX1 axis may regulate their target expression by altering mRNA stability. To test this hypothesis, we focused on the three common targets of the *DARS1-AS1*/YBX1 axis, *E2F1*, *CCND1*, and *FOXM1*, that harbored YBX1 binding sites based on eCLIP-seq data (Fig. 9D). We performed RNA pull-down or RIP followed by RT-qPCR to confirm that they were bound to both YBX1 (Fig. 9, E and F, and fig. S6, L and M) and *DARS1-AS1* (Fig. 9G). We observed that depletion of *DARS1-AS1* reduced YBX1 binding to these target mRNAs after controlling for the target expression change (Fig. 9, H and I), indicating that *DARS1-AS1* promotes YBX1 binding to these targets. To determine the role of *DARS1-AS1*:YBX1 interaction in regulating YBX1 binding to their common target mRNAs, we performed rescue experiments for the decreased YBX1 binding to *E2F1*, *CCND1*, and *FOXM1* upon *DARS1-AS1* knockdown, by overexpressing the full-length siRNA-resistant *DARS1-AS1* RNA (NR_1100199.1) or the mutant RNA (deletion of 1 to 300 bp) with defective interaction with YBX1. We found that overexpression of the full-length siRNA-resistant *DARS1-AS1* rescued the decreased YBX1 binding to their common target mRNAs upon *DARS1-AS1* knockdown, whereas the mutant RNA with defective interaction with YBX1 failed to do so (fig. S7, A and B). These findings indicate that *DARS1-AS1*:YBX1 interaction is required for promoting YBX1 binding to their common targets.

To investigate whether *DARS1-AS1*/YBX1 regulates target expression through YBX1 binding sites in the target 3′UTR, we generated reporter plasmids by fusing the 3′UTR sequence with/without the eCLIP-seq–identified YBX1 binding sites from *FOXM1*, *E2F1*, and *CCND1*, to the 3′ end of firefly luciferase coding sequence (Supplementary Methods), followed by measuring the luciferase activity in the presence/absence of *DARS1-AS1*/*YBX1* knockdown. We found that the luciferase fused to the mutant 3′UTR with deletion of YBX1 binding sites showed a significant reduction in its normalized activity compared with the one fused to the 3′UTR with intact YBX1 binding sites (fig. S7C). Moreover, knockdown of *DARS1-AS1* or *YBX1* significantly reduced the normalized activity of the luciferase fused to the 3′UTR with intact YBX1 binding sites but did not affect the activity of the luciferase fused to the mutant 3′UTR without YBX1 binding sites (fig. S7C). These results demonstrated that the YBX1 binding sites in the 3′UTR were important for mediating *DARS1-AS1*/YBX1 regulation of target expression. To assess the effect of inhibiting *DARS1-AS1* or *YBX1* expression on the stability of the target mRNAs, we measured the decay of the target mRNAs using RT-qPCR following transcriptional inhibition induced by actinomycin D in GBM cells/GSCs. Consistent with our hypothesis that the *DARS1-AS1*/YBX1 axis may regulate their target expression by altering mRNA stability, we found that reducing the expression of *DARS1-AS1* or *YBX1* accelerated the decay (i.e., decreased the stability) of their target mRNAs (Fig. 9, J to O).

As *DARS1-AS1* and YBX1 formed a complex to co-regulate target gene expression, we next sought to identify the potential common regulators of *DARS1-AS1* and YBX1, by systematic computational analysis of the chromatin immunoprecipitation sequencing (ChIP-seq) datasets curated in the Cistrome data browser (*49*), to identify the transcriptional regulators that have at least one ChIP-seq peak within the upstream 2000 bp and downstream 500 bp of the TSS of *DARS1-AS1* or YBX1 (Materials and Methods). We identified 108 and 64 candidate regulators for *DARS1-AS1* and YBX1, respectively, 27 of which were common ones (fig. S7D and table S6). We then randomly selected and experimentally tested 7 of the 26 candidates, excluding the RNA polymerase II subunit A, a general transcriptional regulator (fig. S7, E to G). MED1 and ETS1 were the only two candidates with at least one siRNA, whose transfection led to more than 1.5-fold reduction in RNA expression of both *DARS1-AS1* and *YBX1* (fig. S7, E to G) in U251 cells. This finding suggests that MED1 and ETS1 may be common upstream transcriptional regulators of *DARS1-AS1* and *YBX1*.

## DISCUSSION

The lack of effective treatment of GBM underscores the importance and urgent need of a better understanding of the molecular mechanisms underlying the GBM malignancy. Evidence indicates that GSCs, a subpopulation of highly tumorigenic GBM cells, are capable of self-renewal and contribute to intratumoral heterogeneity. GSCs play an import role in mediating GBM growth, invasion, recurrence, and therapeutic resistance to chemotherapy and radiation therapy. Therefore, understanding the molecular mechanisms that control the functional properties of GSCs may reveal new opportunities for therapeutic interventions. Studies of transcriptional regulatory circuits in GSCs identified the neurodevelopmental transcription factors that are key to GSC tumorigenicity and maintenance (*5*). Despite advances in the understanding of transcriptional regulation mechanism governing the functional characteristics of GSCs, the posttranscriptional regulatory circuits critical for tumorigenesis or chemo-/radio-resistance of GSCs remain poorly understood. Moreover, it is unclear how the posttranscriptional and transcriptional circuits are interconnected and coordinated in controlling the malignance properties of GSCs.

By combining CRISPRi screens with large-scale analysis of molecular/clinical data, we identified the lncRNA genes with clinically relevant expression that may be critical for promoting GBM cell fitness. We used RNAi-mediated knockdown to independently validate the fitness-promoting function of the top-ranked screen hits to control for potential CRISPRi-mediated off-target effect (*23*). We further leveraged orthogonal multiomics data and identified the *DARS1-AS1*/YBX1–controlled core posttranscriptional regulatory programs that stabilize mRNAs of the key regulators of cell cycle progression, self-renewal, and HR-mediated repair of DSBs in GSCs (fig. S7H), underscoring an important and underappreciated role of lncRNA/RBP-mediated posttranscriptional regulatory circuits in governing the malignant features of GSCs. We found that *DARS1-AS1*/YBX1 up-regulated *E2F1* and *CCND1* expression by stabilizing their mRNAs. It was previously shown that *CCND1* is up-regulated by *E2F1* (*50*). In addition, *E2F1* activates *CCND1* expression in GBM cells, at least partially through transcriptionally inhibiting the expression of microRNA-107 that is a posttranscriptional repressor of *CCND1* (*51*). Therefore, *DARS1-AS1*/YBX1, *E2F1*, and *CCND1* formed a unique architect of mixed transcriptional/posttranscriptional feed-forward loop (FFL) (*52*, *53*) to promote the G_1_-S transition. Previous studies revealed that transcriptional regulatory networks contain a small set of recurring regulatory interaction patterns, the so-called network motifs (*52*, *54*). Network motifs often show unique regulatory capacities (*53*) and serve as functional building blocks of the transcriptional regulatory networks. One of the important and frequently occurring motifs in transcriptional regulatory networks in both prokaryotes and eukaryotes is the coherent FFL (*55*). A coherent FFL is composed of three genes: a regulator, X, which activates Y, and gene Z, which is activated by both X and Y. It has a unique regulatory capacity and is an important feature for transcriptional regulatory networks that control the gene expression in human pancreas, liver, and embryonic stem cells (*56*, *57*). Our finding of a coherent FFL controlling G_1_-S transition of GSCs that is composed of both transcriptional and posttranscriptional regulation indicated the importance of the cross-talk between different layers of gene regulation, suggesting that the mixed transcriptional/posttranscriptional FFL is an important regulatory module for controlling the malignancy of GSCs. It remains to be determined what is the unique regulatory capacity of the coherent FFLs with mixed transcriptional/posttranscriptional regulation, in comparison with the ones with pure transcriptional or posttranscriptional regulation, and, as functional regulatory modules, how prevalent they are in the gene regulatory circuits.

The current study focused on the role of the *DARS1-AS1*/YBX1 axis in modulating target mRNA stability (fig. S7H). As YBX1 has been suggested to play diverse roles in gene regulation, including transcription, splicing (*31*, *32*), and translation (*35*, *36*), the role of the *DARS1-AS1*/YBX1 axis in other aspects of gene regulation in GBM remains to be investigated. We demonstrated that *DARS1-AS1* promoted YBX1 binding to their key common targets, and this regulation critically depended on *DARS1-AS1*:YBX1 interaction. Moreover, *DARS1-AS1* not only interacted with the CSD, the canonical RNA binding domain of YBX1, but also the CTD of YBX1, which is known to be important for YBX1 oligomerization in solution and increasing the affinity of the CSD to DNAs/RNAs (*58*–*61*). Previous studies showed that YBX1 may form oligomers in solution (*60*, *61*) and in cells (*59*). Together, it is possible that the interaction between *DARS1-AS1* and the CTD might promote YBX1 engagement with common target RNAs by increasing their affinity to the CSD of YBX1 and/or facilitating the formation of YBX1 oligomers to increase avidity, which awaits further investigation. Because YBX1 is highly expressed in GBM, we expect that the *DARS1-AS1*/YBX1 axis only regulates a subset of YBX1 targets. It remains to be determined what are the targets that are co-bound by *DARS1-AS1* and YBX1 and what are the sequence/structural features of these targets that differentiate them from the other YBX1 targets.

The DNA damage response pathways can activate distinct repair mechanisms, including mismatch repair (MMR), base excision repair (BER), nucleotide excision repair (NER), HR, and nonhomologous end joining (NHEJ), to repair different types of DNA lesions to preserve genome integrity. Previous studies suggest that YBX1 may be involved in BER and MMR, by interacting with DNA repair proteins (*62*, *63*). In contrast, our study revealed a role of YBX1 in promoting HR-mediated DSB repair via a posttranscriptional regulatory mechanism, whereby it stabilizes the mRNAs of the key regulators of HR-mediated DNA repair. This finding suggests that YBX1 may contribute to different DNA repair processes through diverse mechanisms.

The aberrant activation of HR-mediated DNA repair pathway helps GBM cells/GSCs to survive the DSBs induced by ionizing radiation and plays a critical role in the therapeutic resistance of GBM to the radiation therapy. Our study indicates that by inhibiting *DARS1-AS1*, GBM cells/GSCs become more sensitive to ionizing radiation, which implies that targeting the *DARS1-AS1*/YBX1 axis could potentially make GBM more susceptible to radiation therapy. In breast and ovarian cancers, germline/somatic mutations in key components of the HR-mediated DSB repair pathway, like *BRCA1/2*, have been known to cause HR deficiency (HRD) in tumors. This can make these tumors vulnerable to poly(adenosine 5′-diphosphate–ribose) polymerase inhibitor (PARPi) treatment, which has shown clinical efficacy in tumors with *BRCA1/2* mutations (*64*, *65*). On the basis of these findings, we propose that inhibiting the *DARS1-AS1*/YBX1 axis may lead to HRD in GBM and make it vulnerable to PARPi therapy. Combining delivery of siRNAs/miRNA mimics that inhibit the *DARS1-AS1*/YBX1 axis using nanoparticle platforms (*66*) with radiation or PARPi therapy may provide a new therapeutic strategy for treating GBM, which warrants further research.

## MATERIALS AND METHODS

### CRISPRi sgRNA design, lncRNA selection, and library construction

We integrated the FANTOM5 CAGE data with GENCODE V22 transcriptome annotation to define the 5′ ends (i.e., TSSs) of all the transcripts, as described previously (*17*, *19*). It has been demonstrated that integration of CAGE data with GENCODE transcript models improves the accuracy of 5′ end/transcription initiation site annotation of lncRNAs (*17*). Because the effect of the CRISPRi-mediated transcriptional silencing is sensitive to the precise distance from the transcription initiation sites, an accurate annotation of 5′ end/transcription start sites of lncRNAs is critical for designing sgRNAs for effective CRISPRi-mediated repression. The sequences from the 500 bp windows centered on the defined 5′ ends were extracted for sgRNA design, using the SSC method. The designed sgRNAs that meet one of the following criteria: (i) being mapped to multiple genomic regions, (ii) with any Ns or more than three consecutive T, and (iii) with extreme level of GC content (≥75% or <10%), were filtered out. If several sgRNAs were within 4 bp from each other, only the one with the best SSC scores were selected. At gene level, up to eight top-ranked sgRNAs were selected from the corresponding CAGE-defined 5′ end. If multiple CAGE clusters were assigned to a given gene, the sgRNAs were preferably selected from the CAGE clusters with a higher transcription initiation evidence score (TIEScore).

To target the lncRNA genes that showed clinically relevant expression in CRISPRi screens, differential gene expression analyses between GBM and LGG or normal brain tissues were conducted, using RNA-seq data. The RNA-seq data of normal brain tissues and the TCGA RNA-seq data of LGG and GBM tissues were obtained from Gene Expression Omnibus (GSE62098, GSE53239, and GSE59612) and the Genomic Data Commons Data Portal (https://portal.gdc.cancer.gov/), respectively. The raw RNA-seq reads were mapped to the hg38 genome and GENCODE V22 transcriptome annotation using HiSAT2 (*67*) with parameters “--no-discordant --no-mixed.” The differentially expressed lncRNA genes between GBM and LGG or normal brain tissues were identified by edgeR (*68*) (3.24.3) with the filters of |log_2_fold change| ≥ log_2_(1.5) and FDR *<* 0.05 and were considered as GBM-associated lncRNAs. We further filtered out the lowly expressed lncRNA genes (FPKM < 0.5) in all three GBM cell lines U87, U251, and LN229, based on the RNA-seq data from the Cancer Cell Line Encyclopedia project. In the CRISPRi sgRNA library, there are 9083 sgRNAs targeting 1209 GBM-associated lncRNA genes, 835 positive control sgRNAs targeting common essential genes, and 350 *AAVS1* region-targeting sgRNAs/nontargeting sgRNAs that served as negative controls.

The sgRNA library construction was conducted as described previously (*19*). The flanking linker sequences (5′ linker: CTTTATATATCTTGTGGAAAGGACGAAACACCG; 3′ linker: GTTTTAGAGCTAGAAATAGCAAGTTAAAATAAGGCTAGTCCG) were added to each designed sgRNA sequence for library construction. The oligonucleotides containing both sgRNAs and flanking linker sequences were synthesized as a pooled library using the CustmoArray 12K chips (CustmoArray Inc.). The array-synthesized sgRNA library was amplified for eight cycles (primer sequences in table S2) with Q5 High-Fidelity DNA Polymerase (New England Biolabs, #M0491S). The PCR product was purified from 2% agarose gel with the QIAquick Gel Extraction Kit (QIAGEN, #28704). Gibson Assembly (Gibson Assembly Master Mix, New England Biolabs, #E2611L) was used to assemble the amplified sgRNA library into a Bsm BI (Thermo Fisher Scientific, #ER0452)–digested lentiGuide-Puro vector (Addgene, #52963). A total of 2 μl of the product (10 to 50 ng/μl) from Gibson Assembly reaction was added to one tube of 25 μl of electrocompetent cells (Lucigen) on ice for 5 min (~3 to 4 reactions for one library). Electroporation was then conducted using Micropulser Electroporator (Bio-Rad) by the one-shot EC1 program. The transformed electrocompetent cells were recovered in recovery medium and rotated at 250 rpm for 1 hour at 37°C. One milliliter of transformation was plated on each of premade 24.5 cm^2^ bioassay plates (ampicillin) using a spreader. All plates were grown inverted for 14 hours at 32°C. Last, the colonies were scraped off and the plasmids were extracted with a NucleoBond Xtra Midi EF kit (Takara, #740422.50) for downstream virus production. The coverage and distribution of the sgRNA library were evaluated by next-generation sequencing (NGS).

### CRISPRi screen and data analysis

U251 and U87 cells were infected with lentiviruses containing pHR-SFFV-dCas9-BFP-KRAB (Addgene, #46911), and BFP-positive cells were sorted by fluorescence-activated cell sorting (FACS) to generate the cell lines with stable expression of dCas9-BFP-KRAB (U251-dCas9 and U87-dCas9). Lentiviruses containing the sgRNA library were generated by cotransfection of pCMV-VSV-G, psPAX2, and sgRNA library plasmids into human embryonic kidney (HEK) 293FT cells. The cell supernatant containing lentiviruses was collected 48 hours after the transfection, and the infectious titration was conducted in U251-dCas9 and U87-dCas9 cells to determine the lentiviral titer to achieve a multiplicity of infection (MOI) between 0.2 and 0.3. A total of 2.5 × 10^7^ U251-dCas9 or U87-dCas9 cells were plated onto ten 10 cm dish and were infected with lentiviruses containing the sgRNA library at an MOI of 0.2 to 0.3 and 500× coverage for each cell line. The cells transduced with sgRNA library were selected with puro (2 μg/ml) for 4 days and were then split into three replicates. For each replicate, 1 × 10^7^ cells were harvested for genome extraction as D0 control samples. The remaining cells were passed every 3 days and cultured for 21 days. At D21, 1 × 10^7^ cells were harvested for the genome extraction using the QIAamp DNA Mini Kit (QIAGEN) for each replicate. Two rounds of PCR were used to prepare the NGS-ready sgRNA libraries with the KAPA HiFi HotStart ReadyMix (Roche, #KK2602). For each replicate at D0 or D21, 40 μg of input genomic DNA was extracted and used as templates in eight reactions (5 μg per reaction) to conduct the first-round PCR for 16 cycles. The PCR product of different reactions was then pooled, and 20 μl of the mixed product was used as a template in one of the two reactions for the second-round PCR. The second-round PCR was conducted for 12 cycles to incorporate Illumina barcode sequences [forward: AATGATACGGCGACCACCGAGATCTACAC<Illumina index 8-nt barcode>ACACTCTTTCCCTACACGACGCTCTTCCGATCTTCTTGTGGAAAGGACGAAACACCG; reverse: CAAGCAGAAGACGGCATACGAGAT<Illumina index 8-nt barcode>GTGACTGGAGTTCAGACGTGTGCTCTTCCGATCTCTACTATTCTTTCCCCTGCACTGTACC]. The final PCR product was purified from 2% agarose gel with the QIAquick Gel Extraction Kit. Concentration of different libraries was determined using the Qubit dsDNA HS (High Sensitivity) Assay Kit (Thermo Fisher Scientific, # Q32851) on a Qubit Fluorometer (Thermo Fisher Scientific). The libraries were pooled with equal proportion for NGS (single-end 75 bp) on an Illumina NextSeq 500 system at the Advanced Technology Genomics Core Facility of the University of Texas (UT) MD Anderson Cancer Center (MDACC). All primer sequences are listed in table S2. MAGeCK (v0.5.9.4) (*20*) was used to calculate the read count of individual sgRNAs in different samples with the following parameters: “mageck count -l lncRNA_CRISPRi_sgRNA_library --control-sgrna lncRNA_CRISPRi_sgRNA_CTRL --norm-method control -n sgrna.count --sample-label D0,D21 --fastq files.fq.” MAGeCK test module was then applied with parameters “mageck test -k sgrna.count -c D0 -t D21 --norm-method control --keep-tmp -n D21_D0 --control-sgrna lncRNA_CRISPRi_sgRNA_CTRL” to identify the sgRNAs that showed a significant negative selection [log_2_(fold change) ≤ −log_2_(1.5), *P* < 0.01, and FDR < 0.05] between D0 and D21.

### Cell culture

HS683, SW1783, U87, U251, and LN229 cells from S.H.’s laboratory and NHA cells from B.H.’s laboratory were cultured in Dulbecco’s modified Eagle’s medium (DMEM; HyClone, #SH30022.01) supplemented with 10% fetal bovine serum (FBS; Gibco, #10437-028) and 1% penicillin/streptomycin (Corning, #30-002-CI). 293FT cells were obtained from the Characterized Cell Line Core Facility of MDACC and cultured in DMEM supplemented with 10% FBS (Gibco, #10437-028) and 1% penicillin/streptomycin. U2OS/DR-GFP cells were from J.C.’s laboratory and were cultured in DMEM supplemented with 10% FBS and 1% penicillin/streptomycin. GSC11 (classical subtype, wild-type IDH1), GSC272 (mesenchymal subtype, wild-type IDH1), GSC295 (proneural subtype, wild-type IDH1), GSC17 (proneural subtype, wild-type IDH1), GSC262 (proneural subtype, wild-type IDH1), and GSC20 (mesenchymal subtype, wild-type IDH1) cells were originally isolated from fresh surgical specimens of human GBM by F.F.L.’s laboratory and were cultured as GBM neurospheres in DMEM/Ham’s F-12 50/50 Mix medium (Corning, #10-090-CV) supplemented with B-27 supplement (Gibco, #17504044), epidermal growth factor (20 ng/ml; STEMCELL Technologies, #78006.1), and basic fibroblast growth factor (STEMCELL Technologies, #78003). The study was approved by the Institutional Review Board of UT MDACC. For two-dimensional culture of GSCs, the culture plate was precoated with the bovine fibronectin protein (R&D Systems, #1030-FN) diluted in phosphate-buffered saline (PBS) (1:300), and GSCs were attached to the coated plate after 12 to 18 hours. All cell lines were maintained in an incubator at 37°C with 5% CO_2._

### Orthotopic intracranial GBM xenograft experiments

A total of 5 × 10^5^ GSC11/GSC17 cells stably transduced with a negative control shRNA or one of the two shRNAs targeting *DARS1-AS1* were intracranially grafted into the *Foxn1^nu/nu^* athymic nude mice (6- to 8-week-old female) that were purchased from MDACC ERO Breeding Core to establish orthotopic GBM tumors (*n* = 9 of 10 for each control/treatment group). The animals were randomly assigned to different experimental groups. The survival experiments were conducted in a 120-day period for GSC11-grafted mice and 80-day period for GSC17-grafted mice, respectively. The mice were euthanized when they reach end points or on day 80/120 after tumor cell injection. The survival analyses were performed using the Kaplan-Meier method and log-rank test. For tumor formation analysis, 30 (or 21) days after GSC11 (or GSC17) cells stably expressing negative control shRNA or one of the two shRNAs targeting *DARS1-AS1* were intracranially implanted into athymic nude mice (*n* = 8 to 10 for each control/treatment group), the mice were humanely euthanized and the mouse brains were harvested, fixed in 10% formalin, and embedded in paraffin. Tumor formation was determined by a histologic analysis of hematoxylin and eosin (H&E)–stained tissue sections. Tumor volume was calculated using the formula *V* = (*L* × *W*^2^)/2, where *L* represents the largest tumor diameter and *W* represents the perpendicular tumor diameter, where *L* and *W* are the tumor’s long axis and short axis, respectively. All mouse experiments were approved by the Institutional Animal Care and Use Committee of UT MDACC (IACUC Study #00001392-RN02).

### CRISPRi/RNAi-mediated gene silencing and cDNA overexpression

For CRISPRi-mediated gene silencing, one negative control and two gene-specific sgRNAs were used for each gene and were cloned into lentiGuide-Puro vector. HEK293FT cells were cotransfected with pCMV-VSV-G, psPAX2, and sgRNA-expressing lentiGuide-Puro plasmid using jetPRIME (Polyplus transfection, #114-15). Lentiviruses were collected 48 hours after transfection and were then used to infect cell lines with stable expression of dCas9-KRAB fusion protein in the presence of polybrene (Sigma-Aldrich, #TR-1003) before puro selection for 4 days. Total RNA was extracted using the RNeasy Mini Kit (QIAGEN, #74104) 10 days after infection, and RT-qPCR was conducted to determine the knockdown efficiency of each gene-specific sgRNA (*19*). For the siRNA-mediated knockdown, one negative control and two predesigned gene-specific siRNAs were purchased from Sigma-Aldrich. All sequences are shown in table S2. All siRNAs were transfected into cells using Lipofectamine RNAiMAX Transfection Reagent (Thermo Fisher Scientific, #13778150). Total RNA and protein was extracted 48 hours after transfection for RT-qPCR and Western blot analysis of knockdown efficiency. For shRNA-mediated knockdown, the negative control and gene-specific shRNA sequences were cloned into PLKO.1 TRC vector. HEK293FT cells were cotransfected with pCMV-VSV-G, psPAX2, and shRNA-expressing PLKO.1 TRC plasmid using jetPRIME. Lentiviruses were collected 48 hours after transfection and were then used to infect GBM cells or GSCs in the presence of polybrene, before puro selection for 4 days. Total RNA and protein were collected 4 days after infection. RT-qPCR and Western blot were conducted to determine the efficiency of shRNA-mediated knockdown at RNA and protein level. For overexpression of *DARS1-AS1* transcript (NR_110199.1), its complementary DNA (cDNA) was PCR-amplified using the cDNA pool generate from total RNA of U251 cells and cloned into the GATEWAY pENTRY vector (Invitrogen, #A10464), followed by a Gateway LR reaction (Invitrogen, #11791100) to move *DARS1-AS1* cDNA into the lentiviral vector lincXpress backbone (a gift from J. Rinn’s laboratory) that was made by modifying the pLenti6.3/TO/V5-DEST (Snap Gene) destination vector as described previously (*69*). All the sgRNA, siRNA, and shRNA sequences were listed in table S2.

### Reverse transcription quantitative polymerase chain reaction

RT-qPCR was performed as described previously (*19*). Briefly, total RNA was extracted from GBM or GSC cells using the RNeasy Mini Kit (QIAGEN, #74104), according to the manufacturer’s instructions. RNA concentration was measured with a NanoDrop spectrophotometer, and 1 g of total RNA was used for the synthesis of cDNA using the iScript Reverse Transcription Supermix (Bio-Rad, #1708841). RT-qPCR was performed using SsoAdvanced Universal SYBR Green Supermix (Bio-Rad, #1725274) in the CFX96 Touch Real-Time PCR Detection System (Bio-Rad) according to the manufacturer’s instructions. The sequences of primers used in this study were listed in table S2. Glyceraldehyde-3-phosphate dehydrogenase (*GAPDH*) was used as an internal control, and the fold change of lncRNA or mRNA expression was calculated using the ΔΔCT method.

### CCK-8, clonogenic assay, and cell cycle analysis

To assess the effect of overexpressing or silencing a given gene on cell growth, 1 × 10^3^ U87, U251, or LN229 cells per well and 2 × 10^3^ GSC cells per well were seeded in 96-well plates, where each treatment condition and time point was in triplicate. For GSCs, the 96-well plate was precoated with bovine fibronectin protein (R&D Systems, #1030-FN) diluted (1:300) in PBS (Corning, #MT21040CV) and GSCs attached to the coated plate after 12 to 18 hours. From the following day (day 0) to 4 days afterward, cell growth was assessed using Cell Counting Kit-8 (CCK-8) assay (Dojindo Molecular Technologies Inc., #CK04-13). Briefly, 10 μl of CCK-8 (Dojindo Molecular Technologies Inc.) was added into each well and the OD_450_ (optical density at 450 nm) absorbance was measured after 2 hours of incubation at 37°C. For clonogenic assay, 1 × 10^3^ U87, U251, or LN229 cells per well were plated in six-well plates, with each treatment condition in triplicate. The medium was changed every 4 days, and the cells were cultured for 2 weeks. Then, the clones were fixed with methanol and stained with 0.5% crystal violet in PBS. Plates were then washed with distilled water and photographed with ChemiDoc Touch Imaging Systems (Bio-Rad). For cell cycle analysis, cells were fixed with 70% ethanol at −20°C overnight. After washing cells with cold PBS, the pellet was resuspended in 500 μl of PI (Thermo Fisher Scientific, #P1304MP)/Triton X-100 (Sigma-Aldrich, #T8787) staining solution containing deoxyribonuclease (DNase)–free ribonuclease (RNase) A (Thermo Fisher Scientific, #EN0531) and incubated at 37°C for 30 min. The cell cycle distribution of the PI-stained cells was analyzed by flow cytometry at the Flow Cytometry and Cellular Imaging Core Facility of MDACC.

### Self-renewal and soft agar colony formation assay

To determine the self-renewal capacity of GSCs, GSCs cells were dissociated as single cells and seeded in 96-well plates (~1 cell per well). The percentage of wells with formed tumor spheres ≥100 μm in diameter was determined after 10 days of culture. For soft agar colony formation assay, 2 × 10^3^ GSC cells stably transduced with a negative control shRNA or individual *DARS1-AS1*–targeting shRNAs were seeded in a 0.3% low-melting agarose on the top of bottom agar containing 0.5% low-melting agarose per well in six-well plates. With/without x-ray radiation (2.5 Gy) on day 0, after 14 days, the number of colonies ≥50 μm was counted. Irradiation was performed using the X-RAD 320 Biological Irradiator (Precision X-Ray Inc.).

### 5′ and 3′ RACE

The 5′ and 3′ RACE experiments were conducted using the SMARTer RACE 5′/3′ Kit (Clontech, #634859), as described previously (*19*). Briefly, the total RNA of U251 cells was extracted using the RNeasy Mini Kit (QIAGEN, #74104) according to the manufacturer’s instruction. First-strand cDNA was synthesized using 5′-CDS and 3′-CDS primer A and SMARTer II A oligonucleotide as described in the manufacturer’s manual. The touchdown nested PCR was used to amplify cDNA ends. The PCR product was purified from 2% agarose gel with the NucleoSpin Gel and PCR Clean-Up Kit (supplied with the SMARTer RACE 5′/3′ Kit) and was then cloned into pRACE vector using In-Fusion HD Master Mix (both vector and mix were provided as SMARTer RACE 5′/3′ Kit Components). Last, the clones containing the gene-specific inserts were sequenced.

### Nuclear and cytoplasmic fractionation

Nuclear and cytoplasmic RNAs of GBM and GSC cells were isolated using a PARIS kit (Thermo Fisher Scientific, #AM1921) as described previously (*19*), according to the manufacturer’s manual. Briefly, 5 × 10^6^ cells were collected and washed with cold PBS and were then lysed with 500 μl of ice-cold cell fractionation buffer on ice for 10 min. After centrifugation for 5 min (500*g*) at 4°C, the supernatant containing cytoplasmic fraction and the nuclei pellet were collected. The collected nuclei pellet was washed with ice-cold cell fractionation buffer and repelleted by centrifugation for 1 min (500*g*) at 4°C, followed by lysis with cell disruption buffer. The nuclear lysate or the cytoplasmic fraction was mixed with an equal volume of 2× lysis/binding solution and 100% ethanol. The mixture was then transferred to a filter cartridge for RNA purification*. MALAT1* RNA and *GAPDH* mRNA were detected by RT-qPCR in isolated nuclear/cytoplasmic RNAs, as a control for nuclear and cytoplasmic RNA, respectively.

### RNA pull-down and RIP

To systematically identify the proteins that may form physical interaction with *DARS1-AS1*, we adopted an approach that uses the bacteriophage MS2 coat protein (MS2) and its high-affinity cognate RNA binding sites (MS2bs) (PMID: 25240888). The FLAG-tagged MS2 was cloned into pLVX-Puro vector. The lentiviruses containing the FLAG-tagged MS2 were generated and used to infect U251 or GSC11 cells, followed by puro selection for 4 days. Twelve copies of MS2bs were inserted into the pLenti CMV Blast DEST vector near the 3′ end of full-length, mutant, or antisense *DARS1-AS1* RNA. The U251 or GSC11 cells stably expressing FLAG-tagged MS2 were infected with lentiviruses containing MS2bs-tagged wild-type, mutant, or antisense *DARS1-AS1*, followed by blasticidin selection for 4 days. The RNA pull-down experiments were performed as described previously. Briefly, U251 or GSC cells were cross-linked with 1% formaldehyde for 10 min at room temperature and quenched with 250 mM glycine for 5 min, followed by ice-cold PBS wash. The cells were harvested by scraping with a rubber policeman and were pelleted by centrifugation at 1000*g* for 10 min at 4°C. Next, the cell pellet was lysed with sonication in lysis buffer on ice. Cell lysate was precleared with protein G–agarose beads for 1 hour at 4°C. Meanwhile, the anti-FLAG M2 Affinity Gel (Sigma-Aldrich, #A2220) beads was precleared with yeast transfer RNA and was then incubated with precleared cell lysate for 3 hours with gentle rotation at 4°C, followed by centrifugation at 1000*g* for 1 min at 4°C. The beads were then washed 10 times with NET-2 buffer for 2 min each at 4°C, followed by elution with 3XFLAG peptide (Sigma-Aldrich, #F4799) in tris-buffered saline buffer. The cross-links were reversed by heating the sample sequentially for 5 min at 95°C, for 1 hour at 65°C, and for 5 min at 95°C. Half of the sample was used to extract RNA with the RNeasy Mini Kit (QIAGEN, #74104) for RT-PCR analysis, and the other half was used to extract proteins for MS or Western blot analysis.

The RIP assay was conducted following the manufacturer’s manual using an EZ-Magna RIP RBP IP kit (Millipore, #17-701), as described previously (*19*). Briefly, cells in 15 cm plate were washed with ice-cold PBS, scraped off from each plate, and collected by centrifugation at 1500 rpm for 5 min at 4°C. The collected cell pellet was resuspended in an equal pellet volume of complete RIP lysis buffer, incubated on ice for 5 min, and stored at −80°C. Next, the magnetic beads were washed with RIP wash buffer and incubated with antibodies for 30 min at room temperature with rotation. After incubation, the antibody-bead complex was washed twice with RIP wash buffer. Once thawed, the RIP lysate was centrifuged at 14,000 rpm for 10 min at 4°C. One hundred microliters of the supernatant was mixed with the antibody-bead complex in RIP immunoprecipitation buffer, and the mixture was incubated at 4°C for 4 hours with rotation. The beads were washed six times with RIP wash buffer and then incubated with proteinase K at 55°C for 30 min with shaking to digest the protein. Last, RNA was extracted with phenol-chloroform for RT-qPCR analysis.

### RNA-seq, eCLIP-seq, and data analysis

RNA-seq was performed as described previously (*19*). Briefly, total RNA was isolated from U251 cells using the RNeasy Mini Kit (QIAGEN, #74104) and was treated with DNase I (QIAGEN, #79254). Two micrograms of RNA was used for RNA-seq library construction with a TruSeq Stranded mRNA Library Prep kit (Illumina, #20020594). Sequencing of the library (75 bp single-end read) was conducted on the Illumina NextSeq 500 System, at the Advanced Technology Genomics Core Facility of MDACC. The RNA-seq reads were trimmed for adaptor sequence and masked for low-complexity and low-quality sequence. They were then mapped to the hg38 genome and GENCODE V22 transcriptome, using STAR (2.6.1b) (*70*) with parameters “--outSAMunmapped Within --outFilterType BySJout --twopassMode Basic --outSAMtype BAM SortedByCoordinate.” The gene-level raw read counts were calculated using htseq-count function of HTSeq (0.11.0) (*71*) with parameters “--stranded reverse --additional-attr gene_name gene_type.” The normalization of raw read counts and differential gene expression between the treatment and control conditions were identified using DESeq2 (1.22.2) [|log_2_fold change| ≥ log_2_(1.5) and FDR < 0.05]. GSEA was performed on the RNA-seq data generated in the U251 cells with/without siRNA-mediated *DARS1-AS1* knockdown using GSEA (v4.2.2) and the curated gene sets (C2) from the Molecular Signatures Database (MSigDB v6.2) (https://gsea-msigdb.org/gsea/index.jsp). The gene sets with FDR≤0.1 were considered as being enriched (table S4). The GO enrichment analysis was performed by DAVID (https://david.ncifcrf.gov/).

The eCLIP libraries were generated from U251 cells in biological duplicates with the kit (Eclipse Bioinnovations Inc., #ECEK-0001) according to the standard single-end eCLIP protocol. Briefly, 2 × 10^7^ U251 cells stably expressing FLAG-tagged YBX1 for each replicate were cross-linked by 254 nm UV light (400 mJ/cm^2^) and snap-frozen. In the following day, the cell pellet from −80°C was lysed with eCLIP lysis mix and sonicated at 4°C using a Diagenode Bioruptor to disrupt chromatin and fragment DNA. Lysate was treated with RNase I to fragment RNAs. RBP-RNA complex were immunoprecipitated overnight at 4°C, using the Protein G Dynabeads (Thermo Fisher Scientific, #10004D) prebound to an anti-FLAG antibody (Sigma-Aldrich, #F1804). Two percent of the whole-cell lysate was saved as size-matched input (SMInput) samples and run alongside with IP samples. IP samples were subjected to a series of stringent washes, and for all samples, the RNA was dephosphorylated with FastAP and T4 PNK, followed by on-bead ligation of barcoded RNA adapters to the 3′ end, according to the manufacturer’s instructions. The immunoprecipitated RBP-RNA complexes were eluted from the beads and were run on standard polyacrylamide gels and transferred to nitrocellulose membranes where the RNA in the region ~45 to ~120 kDa was excised off the membrane and treated with proteinase K. Immunoprecipitation and input was confirmed by parallel Western blotting of fractions of each sample with the antibody described previously. RNA was then reverse-transcribed, and the 3′ ends of the resultant cDNA were ligated to a DNA adaptor, followed by PCR amplification to generate the sequencing libraries, according to the manufacturer’s instructions. The libraries were sequenced on an Illumina HiSeq2500 (single-end 50 bp) at the Avera Institute for Human Genetics. The eCLIP (v0.7.1) (*48*) pipeline was used to analyze eCLIP-seq data with the default parameters on hg38 reference genome to call the significant peaks (*P* < 0.05 and fold change > 1.2). The significant peaks were sorted by the log_2_fold change, and the top 2000 peaks were used for the de novo motif analysis with the HOMER (v4.11-2) (*72*) motif finding script: findMotifsGenome.pl eCLIP.peaks.sig.top2000.bed hg38 motif.analysis.out/ -norevopp. CTK’s (*73*) script “bed2annotation.pl” was used to generate the genomic distribution for the YBX1 eCLIP-seq peaks with default parameters on “hg38” ref-genome.

### GBM molecular subtype and survival analysis

The RNA-seq dataset “TCGA TARGET GTEx” was downloaded from UCSC Xena (http://xena.ucsc.edu/). The normalized *DARS1-AS1* expression data in the GBM tumors corresponding to the mesenchymal, classical, and proneural subtype (*25*) and the normal brain tissues from GTEx were extracted with a custom script for differential expression analysis. The “wilcox.test” from R package “stats” was applied for the significance evaluation of the *DARS1-AS1* expression among different sample types. The log-rank test and the Kaplan-Meier method were used for analyzing the association between *DARS1-AS1* expression and GBM patient overall survival (R packages “survival” and “survminer”). Multivariate Cox proportional hazards regression analysis was performed using the “coxph” from R package “survival” and included the covariates of *DARS1-AS1* expression, sex, age, and GBM subtype. The samples with “NA” for any of the covariates were excluded from Cox regression analysis.

### Immunofluorescence analysis of γ-H2AX foci

U251 and GSC11 cells were seeded on coverslips with 50 to 70% confluence and were then washed with cold PBS, fixed with 4% paraformaldehyde for 10 min, and permeabilized with 0.5% Triton X-100 solution for 10 min on ice. Samples were blocked with 3% bovine serum albumin in PBS for 30 min, incubated with the γ-H2AX primary antibody (Cell Signaling Technology, #9718) overnight at 4°C, and washed three times with PBS for 5 min each. They were then incubated with Alexa Fluor 488–conjugated goat anti-rabbit immunoglobulin G (IgG) (H+L) highly cross-adsorbed secondary antibody (Invitrogen, #A32731) for 2 hours at room temperature in the dark, followed by rinsing three times with PBS for 5 min each. Coverslips were mounted on the microscope slides with Vectashield Mounting Medium containing 4′,6-diamidino-2-phenylindole (DAPI) (Vector Laboratories, #H-1200-10) and analyzed using a Leica SP8 confocal microscope to quantitate the number of nuclei positive for foci at the Advanced Microscopy Core Facility of MDACC. The cells with more than five foci in the nucleus were considered as γ-H2AX foci–positive ones.

### The DR-GFP reporter assay

The U2OS cells harboring the DR-GFP reporter were transfected with pCBASce I–Sce I expression plasmid or empty plasmid using jetPRIME (Polyplus transfection, #114-15) in six-well plates. Twelve hours after transfection, cells were transfected with the siRNAs targeting *DARS1-AS1*, *YBX1*, or the negative control siRNAs using Lipofectamine RNAiMAX Transfection Reagent (Thermo Fisher Scientific, #13778150). After 48 hours, the cells were trypsinized, washed with PBS, fixed with 4% paraformaldehyde, and resuspended in PBS. The percentage of GFP-positive cells was quantitated by flow cytometry at the Flow Cytometry and Cellular Imaging Core Facility of MDACC. To perform DR-GFP reporter assay in U251 cells, U251 cells were transfected with linearized pHPRT-DRGFP (Addgene #26476) and selected with puro (2 μg/μl) for 3 days. The puro-resistant U251 clones were pooled, and the reporter assay was performed as described for U2OS cells.

### Western blot analysis

Western blot analysis was performed as described previously (*19*). Briefly, total protein extract was prepared from the cultured cell lines using radioimmunoprecipitation assay lysis and extraction buffer (Thermo Fisher Scientific, #89900) supplemented with protease inhibitor cocktail (Sigma-Aldrich, #11697498001). The concentration of total protein was quantitated using the Bradford dye-binding method (Bio-Rad, #5000006). Twenty micrograms of protein was loaded and separated by 4 to 15% Mini-PROTEAN TGX precast polyacrylamide gel (Bio-Rad, #4561085) and then transferred to 0.22 μm polyvinylidene fluoride (PVDF) membranes (Millipore, #ISEQ00010). PVDF membranes were blocked with 5% nonfat milk and incubated with specific antibodies for detecting different proteins (see detailed information about antibody information in table S2). After the blot is incubated in enhanced chemiluminescence chromogenic substrate (Millipore, #WBKLS0100), protein bands were detected by the ChemiDoc Touch Imaging System (Bio-Rad), and the signal was quantified using Image Lab software (Bio-Rad).

### Statistical analysis

When applicable, the data are presented as the mean ± SD. The two-tailed Student’s *t* test or Wilcoxon rank sum test was used for the comparisons between two groups, and one-way analysis of variance (ANOVA) with Dunnett’s or Tukey’s multiple comparison test was used for more than two groups, using GraphPad Prism 9.0 or R package. Log-rank test or multivariate Cox proportional hazards regression analysis was used for survival analysis. The numbers of animals or replicates used to perform the experiments are indicated in the figure legends. Data in RT-qPCR analysis are presented as technical triplicates. RT-qPCR and Western blot data are representative of three independent experiments. No statistical methods were used to predetermine sample size. *P* values of less than 0.05 were considered to be statistically significant in all cases. There were no blinding events for in vivo studies.
