# Supplementary material for: Multiomics analyses reveal DARS1-AS1/YBX1–controlled posttranscriptional circuits promoting glioblastoma tumorigenesis/radioresistance
Source: Sci Adv. 2023 Aug 4;9(31):eadf3984. doi: 10.1126/sciadv.adf3984 (PMC10403220; doi:10.1126/sciadv.adf3984)
Supplement: Supplementary file 1 — Supplementary Methods Figs. S1 to S7 Legends for tables S1 to S6 [file sciadv.adf3984_sm.pdf]

Science Advances
AAAS

Supplementary Materials for

Multiomics analyses reveal *DARS1-AS1*/YBX1–controlled posttranscriptional circuits promoting glioblastoma tumorigenesis/radioresistance

Caishang Zheng *et al.*

Corresponding author: Yiwen Chen, ychen26@mdanderson.org, ywcus08@gmail.com; Ming Sun, sunming348@hotmail.com

Sci. Adv. **9**, eadf3984 (2023)
DOI: 10.1126/sciadv.adf3984

The PDF file includes:

Supplementary Methods
Figs. S1 to S7
Legends for tables S1 to S6

Other Supplementary Material for this manuscript includes the following:

Tables S1 to S6

Supplementary Methods.

In vitro RNA pull-down

To perform in vitro RNA pull-down assay for DARS1-AS1 (NR_110199.1), we used the Pierce™ Magnetic RNA-Protein Pull-Down Kit (Thermo Fisher #20164) and adopted a published protocol, as described previously (PMID: 27659978). Briefly, the full-length DARS1-AS1 cDNA sequence was PCR-amplified with an addition of T7 promoter sequence to its 5′ end (see primer sequences in Table S3). T7 RiboMAX™ Express Large-Scale RNA Production System (Promega #P1320) was used to produce full-length RNAs by in vitro transcription with the PCR-amplified T7-promoter-cDNA template, according to the manufacturer's instruction. Transcribed RNAs were purified using RNeasy Mini Kit (QIAGEN, # 74004) and desthiobiotin-labeled using Pierce RNA 3′ End Desthiobiotinylation Kit (Thermo Fisher, # 20163). A total of 50 pmol desthiobiotin-labeled RNA was incubated with 50 µL streptavidin magnetic beads for 30 min at room temperature with agitation. RLN buffer and protein lysis buffer were used to prepare cell extract from the U251 cells that were transfected with the vectors expressing GFP or GFP-tagged full length and truncation mutant YBX1. The streptavidin magnetic beads were then washed twice with an equal volume of 20 mM Tris buffer and incubated with prepared cell extract in protein-RNA binding buffer at 4 °C with agitation or rotation for 1 h. After washing 4 times with wash buffer, the RNA-binding protein complexes were eluted with elution buffer and analyzed with semi-quantitative RT-PCR or western blot.

Dual luciferase reporter assay

The wild-type 3'UTR of E2F1, CCND1, and FOXM1, or their mutant 3'UTR with deletion of YBX1 binding sites were synthesized (Twist Bioscience) and inserted downstream of the firefly luciferase transcriptional unit of pGL3 firefly luciferase reporter vector (Promega, #E1761). The pRL-TK vector (Promega, #E2241) was used as Renilla luciferase control reporter. The U251 cells were co-transfected with the reporter plasmid containing firefly luciferase fused to wild type or mutant 3'UTR sequences, pRL-TK plasmid (as internal control) and the negative control siRNA (siNC)/siRNA targeting DARS1-AS1/YBX1 for 24 h. The cells were then plated in 96-wells plate and cultured for another 48 h before being lysed for measuring luciferase activity. Dual luciferase reporter assay was performed using the Dual-Glo® Luciferase assay kit (Promega, #E2940), according to the manufacturer's instructions. Briefly, cells in 96-wells plate were incubated with 75 ul Dual-Glo regent for at least 10 min at room temperature. The plate was then loaded to the Synergy™ H1 Microplate Reader for firefly luciferase luminescence measurement. Following the completion of firefly luciferase luminescence measurement, another 75 ul Dual-Glo® Stop & Glo® Reagent was added to each well and incubated for 10 min. The plate was then loaded to the Synergy™ H1 Microplate Reader for Renilla luciferase luminescence measurement. The luminescence was measured with a 10s of integration time and the gain setting at 135. The normalized luciferase activity data are represented as the ratio of firefly to Renilla luciferase luminescence.

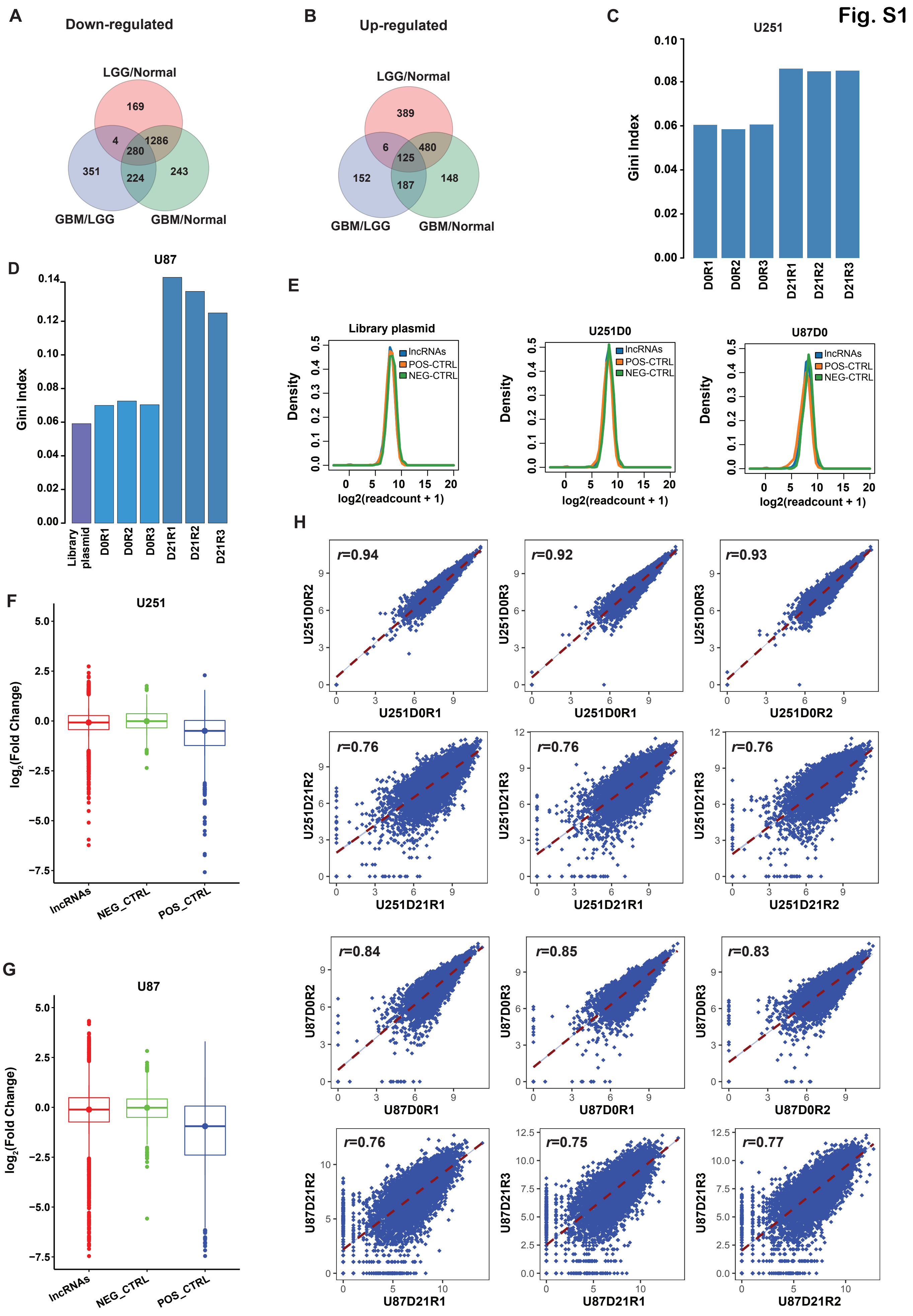
Fig. S1
A
Down-regulated
LGG/Normal
169
4
1286
280
351
224
243
GBM/LGG
GBM/Normal
B
Up-regulated
LGG/Normal
389
6
480
125
152
187
148
GBM/LGG
GBM/Normal
C
U251
Gini Index
D0R1
D0R2
D0R3
D21R1
D21R2
D21R3
D
U87
Gini Index
Library plasmid
D0R1
D0R2
D0R3
D21R1
D21R2
D21R3
E
Library plasmid
U251D0
U87D0
Density
log2(readcount + 1)
lncRNAs
POS-CTRL
NEG-CTRL
F
U251
log2(Fold Change)
lncRNAs
NEG_CTRL
POS_CTRL
G
U87
log2(Fold Change)
lncRNAs
NEG_CTRL
POS_CTRL
H
r=0.94
r=0.92
r=0.93
U251D0R2
U251D0R3
U251D0R1
U251D0R2
r=0.76
r=0.76
r=0.76
U251D21R2
U251D21R3
U251D21R1
U251D21R2
r=0.84
r=0.85
r=0.83
U87D0R2
U87D0R3
U87D0R1
U87D0R2
r=0.76
r=0.75
r=0.77
U87D21R2
U87D21R3
U87D21R1
U87D21R2

Figure S1. Venn diagrams showing the overlap of the significantly (A) up-regulated or (B) down-regulated lncRNA genes ($|\log_2$Fold-Change$| \geq \log_2(1.5)$, *FDR*<0.05) from the following comparisons: GBM tumors vs. normal brain tissues (normal), low-grade glioma (LGG) vs. normal and GBM vs. LGG. Gini index characterizing the uniformity of the sgRNA coverage in the library plasmid or the libraries prepared from (C) U251 and (D) U87 cells on day 0 (D0) and day 21 (D21). (E) Density plots showing the distribution of the negative control (green)/positive control (orange) sgRNAs or the sgRNAs targeting the lncRNAs (blue) in the library plasmid, and the D0 libraries from U251 and U87 cells. Boxplots showing the $\log_2$(Fold-Change) between day 21 and day 0 for sgRNAs targeting the lncRNAs (red), the negative controls (green), and the positive control genes (blue) in (F) U251 and (G) U87 cells. (H) The scatter plots showing the correlations of sgRNA abundances between the three replicates of D0 and D21 libraries from U251 and U87 cells. The Pearson correlation coefficient was used to quantify the correlations and all the correlations are statistically significant ($P < 2.2 \times 10^{-16}$).

Fig. S2

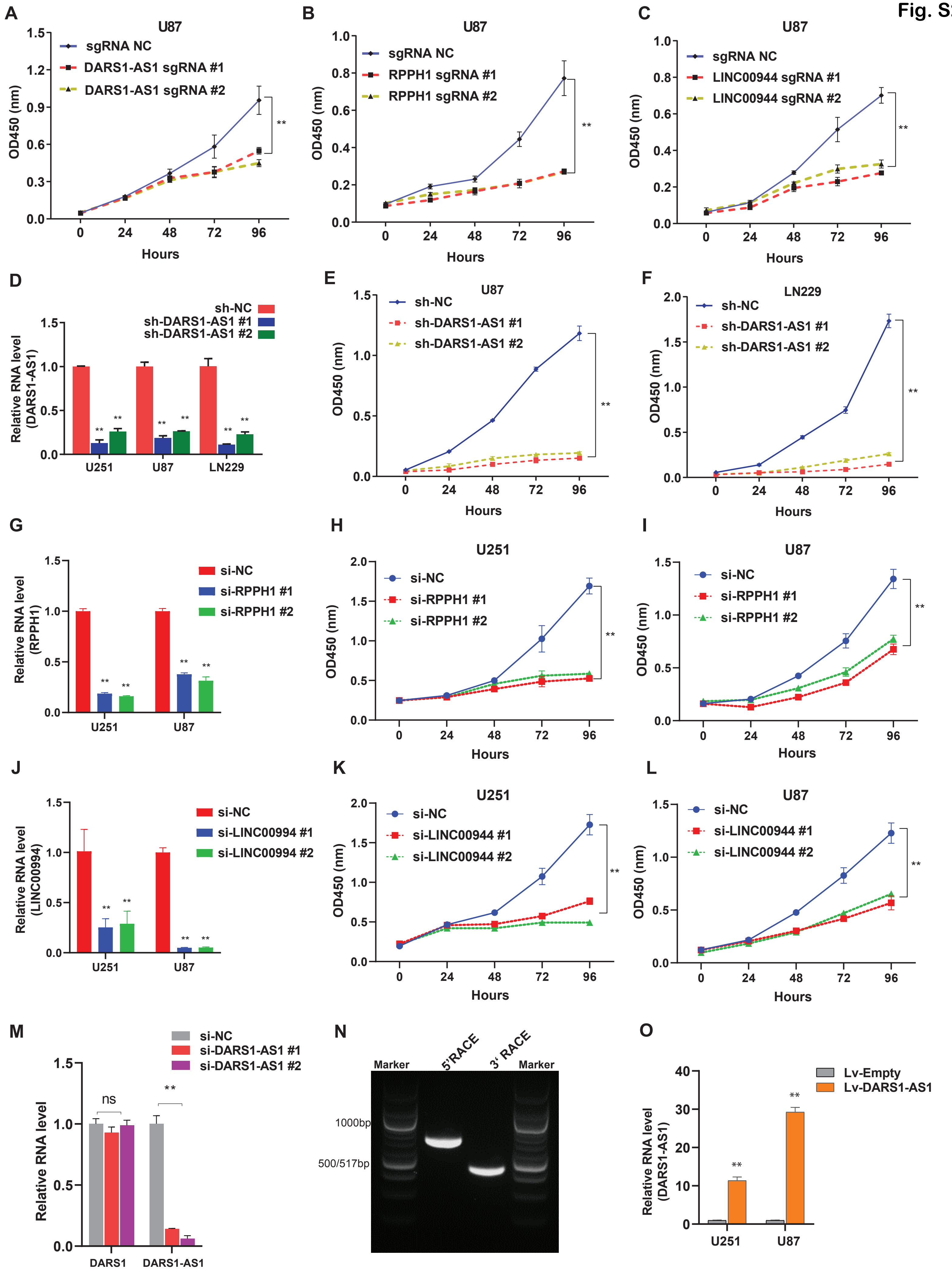

A
U87
sgRNA NC
DARS1-AS1 sgRNA #1
DARS1-AS1 sgRNA #2
OD450 (nm)
Hours
**
B
U87
sgRNA NC
RPPH1 sgRNA #1
RPPH1 sgRNA #2
OD450 (nm)
Hours
**
C
U87
sgRNA NC
LINC00944 sgRNA #1
LINC00944 sgRNA #2
OD450 (nm)
Hours
**
D
sh-NC
sh-DARS1-AS1 #1
sh-DARS1-AS1 #2
Relative RNA level (DARS1-AS1)
U251
U87
LN229
E
U87
sh-NC
sh-DARS1-AS1 #1
sh-DARS1-AS1 #2
OD450 (nm)
Hours
F
LN229
sh-NC
sh-DARS1-AS1 #1
sh-DARS1-AS1 #2
OD450 (nm)
Hours
G
si-NC
si-RPPH1 #1
si-RPPH1 #2
Relative RNA level (RPPH1)
U251
U87
H
U251
si-NC
si-RPPH1 #1
si-RPPH1 #2
OD450 (nm)
Hours
I
U87
si-NC
si-RPPH1 #1
si-RPPH1 #2
OD450 (nm)
Hours
J
si-NC
si-LINC00994 #1
si-LINC00994 #2
Relative RNA level (LINC00994)
U251
U87
K
U251
si-NC
si-LINC00944 #1
si-LINC00944 #2
OD450 (nm)
Hours
L
U87
si-NC
si-LINC00944 #1
si-LINC00944 #2
OD450 (nm)
Hours
M
si-NC
si-DARS1-AS1 #1
si-DARS1-AS1 #2
Relative RNA level
ns
DARS1
DARS1-AS1
N
Marker
5'RACE
3' RACE
Marker
1000bp
500/517bp
O
Lv-Empty
Lv-DARS1-AS1
Relative RNA level (DARS1-AS1)
U251
U87

Figure S2. The growth of the U87 cells transduced with the negative control sgRNA (sg-NC)/sgRNAs targeting (A) DARS1-AS1, (B) RPPH1 or (C) LINC00944 was monitored every 24 h with CCK-8 assay for 96 h. (D) RT-qPCR analysis of the knockdown efficiency for the indicated shRNAs targeting DARS1-AS1 compared with the negative control shRNA (sh-NC) in U251, U87 and LN229 cells, where GAPDH was used as an internal control. The growth of the (E) U87 and (F) LN229 cells transduced with the sh-NC/DARS1-AS1-targeting shRNAs was monitored with CCK-8 assay for the indicated time intervals. (G) RT-qPCR analysis of the knockdown efficiency for the indicated siRNAs targeting RPPH1 compared with the negative control siRNA (si-NC) in U251 and U87 cells. The growth of the (H) U251 and (I) U87 cells transfected with the si-NC/RPPH1-targeting siRNAs was monitored with CCK-8 assay for the indicated time intervals. (J) RT-qPCR analysis of the knockdown efficiency for the indicated siRNAs targeting LINC00994 compared with the si-NC in U251 and U87 cells. The growth of the (K) U251 and (L) U87 cells transfected with the si-NC/LINC00994-targeting siRNAs was monitored with CCK-8 assay for the indicated time intervals. (M) RT-qPCR analysis of DARS1 and DARS1-AS1 RNA expression in the U251 cells transfected with the si-NC or siRNAs targeting DARS1-AS1, where GAPDH was used as an internal control. (N) The 5' and 3' end sequences of DARS1-AS1 were identified by 5' RACE and 3' RACE. The corresponding PCR products were analyzed by agarose gel electrophoresis. (O) RT-qPCR analysis of the DARS1-AS1 RNA level in the U87 and U251 cells transduced with the empty lentiviral lincXpress vector (Lv-Empty) or the lincXpress vector expressing DARS1-AS1 (Lv-DARS1-AS1). Data in **A-M** are shown as mean±standard deviation (SD) (n=3). $^{**}P < 0.01$ or ns, not significant ($P > 0.05$) by 1-way ANOVA with Dunnett's multiple-comparison test. Data in **O** are shown as mean±SD (n=3). $^{**}P < 0.01$ by Student's *t*-test.

Fig. S3

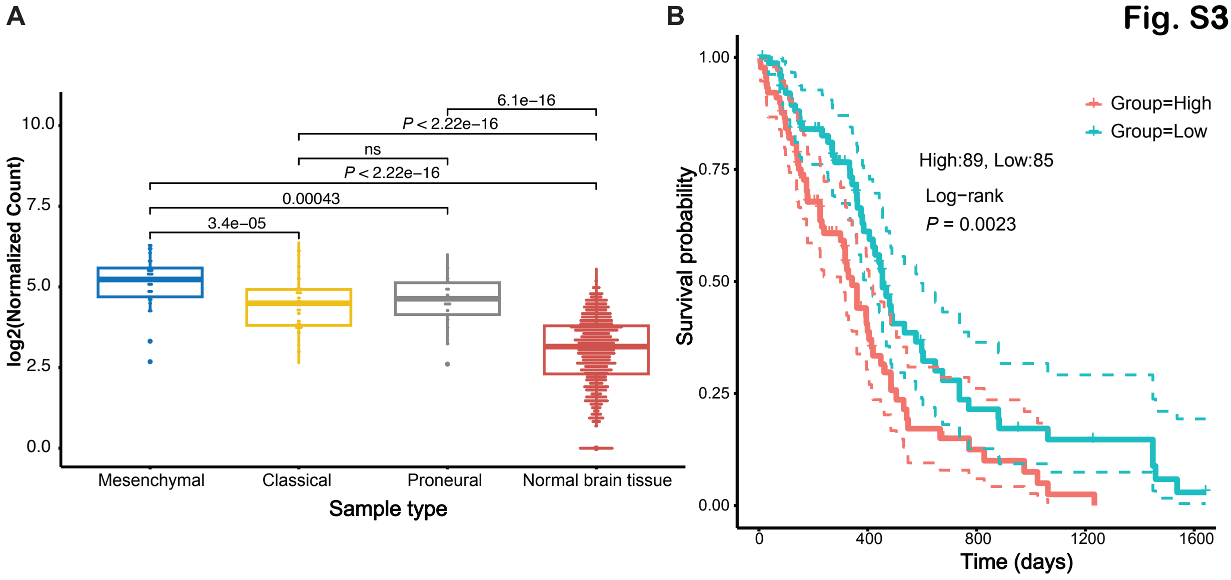
A
6.1e−16
P < 2.22e−16
ns
P < 2.22e−16
0.00043
3.4e−05
10.0
7.5
5.0
2.5
0.0
log2(Normalized Count)
Mesenchymal
Classical
Proneural
Normal brain tissue
Sample type
B
Group=High
Group=Low
High:89, Low:85
Log−rank
P = 0.0023
1.00
0.75
0.50
0.25
0.00
Survival probability
0
400
800
1200
1600
Time (days)

Figure S3. (A) Boxplots showing the RNA expression of DARS1-AS1 in 3 GBM subtypes (Classical, Mesenchymal and Proneural) and normal brain tissues based on TCGA and GTEx data. The statistical significance of the difference between subtypes/normal brain tissues was assessed by Wilcoxon rank-sum test; ns, not significant ($P > 0.05$). (B) Higher DARS1-AS1 expression was associated with shorter overall survival of GBM patients, based on TCGA data. The Kaplan-Meier survival curves are plotted for patient groups with high (top 50%) and low (bottom 50%) DARS1-AS1 expression in GBM tumors. The *P*-value was calculated using log-rank test.

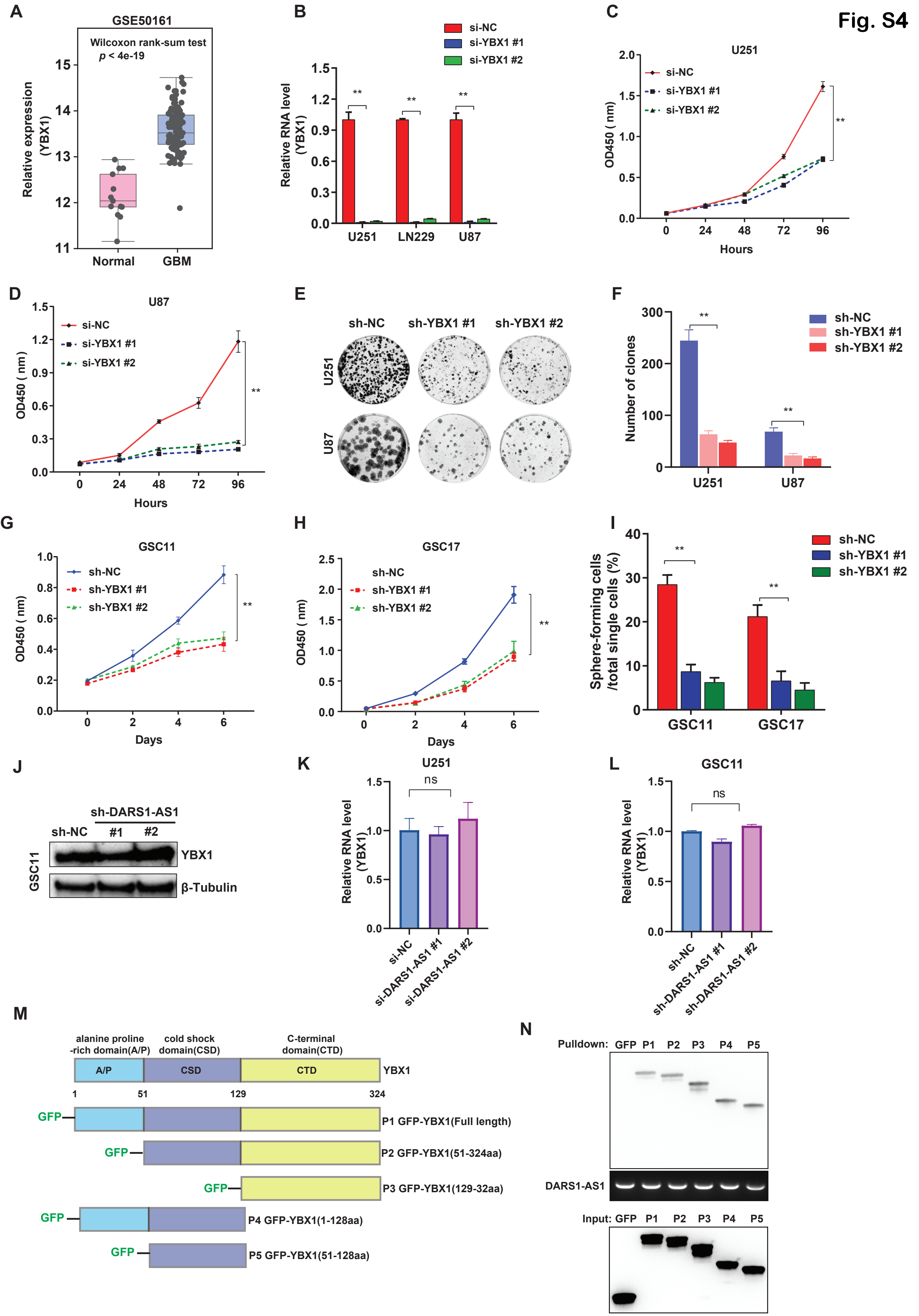

Fig. S4
A
GSE50161
Wilcoxon rank-sum test
p < 4e-19
Relative expression (YBX1)
Normal
GBM
B
si-NC
si-YBX1 #1
si-YBX1 #2
Relative RNA level (YBX1)
U251
LN229
U87
C
U251
si-NC
si-YBX1 #1
si-YBX1 #2
OD450 (nm)
Hours
D
U87
si-NC
si-YBX1 #1
si-YBX1 #2
OD450 (nm)
Hours
E
sh-NC
sh-YBX1 #1
sh-YBX1 #2
U251
U87
F
sh-NC
sh-YBX1 #1
sh-YBX1 #2
Number of clones
U251
U87
G
GSC11
sh-NC
sh-YBX1 #1
sh-YBX1 #2
OD450 (nm)
Days
H
GSC17
sh-NC
sh-YBX1 #1
sh-YBX1 #2
OD450 (nm)
Days
I
sh-NC
sh-YBX1 #1
sh-YBX1 #2
Sphere-forming cells /total single cells (%)
GSC11
GSC17
J
sh-DARS1-AS1
sh-NC
#1
#2
GSC11
YBX1
β-Tubulin
K
U251
ns
Relative RNA level (YBX1)
si-NC
si-DARS1-AS1 #1
si-DARS1-AS1 #2
L
GSC11
ns
Relative RNA level (YBX1)
sh-NC
sh-DARS1-AS1 #1
sh-DARS1-AS1 #2
M
alanine proline -rich domain(A/P)
cold shock domain(CSD)
C-terminal domain(CTD)
A/P
CSD
CTD
YBX1
1
51
129
324
GFP
P1 GFP-YBX1(Full length)
P2 GFP-YBX1(51-324aa)
P3 GFP-YBX1(129-32aa)
P4 GFP-YBX1(1-128aa)
P5 GFP-YBX1(51-128aa)
N
Pulldown: GFP P1 P2 P3 P4 P5
DARS1-AS1
Input: GFP P1 P2 P3 P4 P5

Figure S4. (A) Boxplots showing the RNA expression of YBX1 in pediatric GBM tumors and normal brain tissues based on a published gene expression profiles generated using Affymetrix HG-U133plus2 chips (GSE50161). The statistical significance of the difference in DARS1-AS1 expression between the tumors and normal brain tissues was assessed by Wilcoxon rank-sum test. (B) RT-qPCR analysis of YBX1 RNA expression in the U251, U87 and LN229 cells transfected with the negative control siRNA (si-NC) or siRNAs targeting YBX1, where GAPDH was used as an internal control. The growth of the (C) U251 and (D) U87 cells transfected with si- NC/siRNAs targeting YBX1, were monitored with CCK-8 assay for the indicated time intervals. (E) Representative pictures of clonogenic growth and (F) bar graph quantifying the colonies formed by the U251/U87 cells transduced with the negative control shRNA (sh-NC)/shRNAs targeting YBX1. The growth of the (G) GSC11 and (H) GSC17 cells transduced with sh-NC)/shRNAs targeting YBX1, was monitored with CCK-8 assay. (I) The percentages of GSC11 and GSC17 cells transduced with sh-NC or YBX1-targeting shRNAs that can form neurospheres from single cells determined by self-renewal assay. (J) The protein level of YBX1 was determined by western blot in the GSC11 cells transduced with sh- NC/DARS1-AS1-targeting shRNAs, where β-tubulin was used as a loading control. RT-qPCR analysis of the RNA level of YBX1 in (K) the U251 transfected with si-NC/DARS1-AS1-targeting siRNAs or (L) the GSC11 cells transduced with sh-NC/DARS1-AS1-targeting shRNAs. (M) Schematic diagrams showing different domains of YBX1 and the GFP-tagged full-length or truncation mutant YBX1. (N) RNA pull-down coupled with anti-GFP western blot to detect the interaction between DARS1-AS1 and GFP, GFP-tagged full-length YBX1 or its truncation mutants using the cells that ectopically expressed individual proteins, wherein GFP serves as a negative control. The DARS1-AS1 retrieved from the pull-down was detected by semi-quantitative RT-PCR. Data in **B**-**D**, **F**-**I**, and **K**-**L** are shown as mean±SD (n=3). $^{**}P < 0.01$ or ns, not significant ($P > 0.05$) by 1-way ANOVA with Dunnett's multiple-comparison test.

Fig. S5

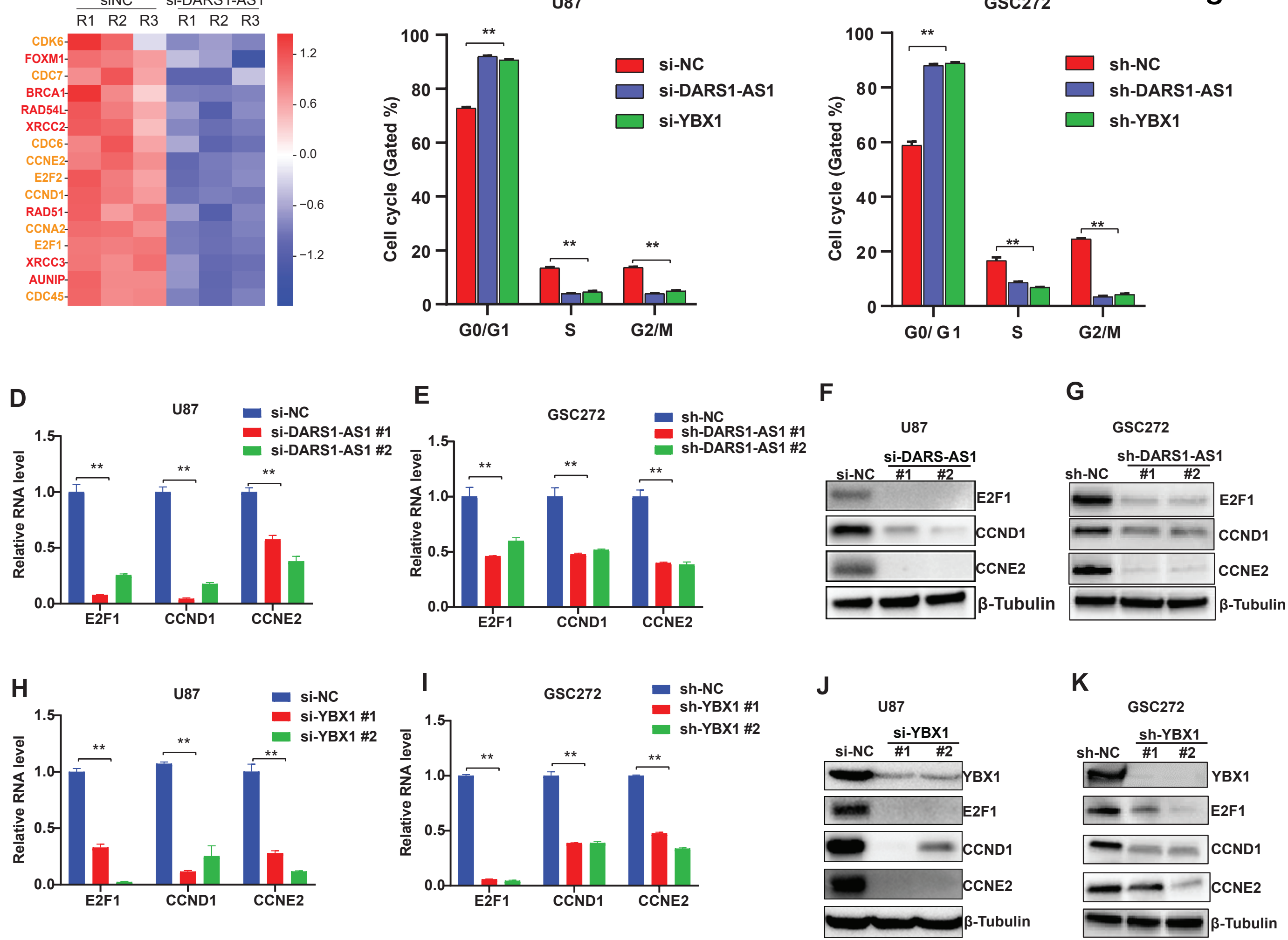

A
siNC
si-DARS1-AS1
R1 R2 R3 R1 R2 R3
CDK6
FOXM1
CDC7
BRCA1
RAD54L
XRCC2
CDC6
CCNE2
E2F2
CCND1
RAD51
CCNA2
E2F1
XRCC3
AUNIP
CDC45
1.2
0.6
0.0
−0.6
−1.2
B
U87
Cell cycle (Gated %)
si-NC
si-DARS1-AS1
si-YBX1
G0/G1
S
G2/M
C
GSC272
sh-NC
sh-DARS1-AS1
sh-YBX1
G0/ G1
S
G2/M
D
U87
Relative RNA level
si-NC
si-DARS1-AS1 #1
si-DARS1-AS1 #2
E2F1
CCND1
CCNE2
E
GSC272
sh-NC
sh-DARS1-AS1 #1
sh-DARS1-AS1 #2
F
U87
si-DARS-AS1
si-NC
#1
#2
E2F1
CCND1
CCNE2
β-Tubulin
G
GSC272
sh-DARS1-AS1
sh-NC
#1
#2
H
U87
si-NC
si-YBX1 #1
si-YBX1 #2
I
GSC272
sh-NC
sh-YBX1 #1
sh-YBX1 #2
J
U87
si-YBX1
YBX1
K
GSC272
sh-YBX1

Figure S5. (A) Heatmap showing the expression of genes up-regulated by DARS1-AS1 and YBX1 (i.e., down-regulated by siRNA-mediated depletion of DARS1-AS1 and YBX1), which are associated with cell cycle (orange) or homologous recombination-mediated DNA repair pathways (red) in the U251 cells transfected with the negative control siRNA (si-NC) or DARS1-AS1-targeting siRNA. Flow cytometry based cell cycle analyses were performed for the (B) U87 and (C) GSC272 cells that were transfected/transduced with si-NC/the negative control shRNA (sh-NC) or siRNAs/shRNAs targeting DARS1-AS1 and were stained with propidium iodide. The percentages of the cells in the indicated cell cycle stages were quantified and shown in the bar graph. RT-qPCR analysis of the RNA level of the established regulators of cell cycle E2F1, CCND1 and CCNE2 in the (D) U87 and (E) GSC272 cells transfected/transduced with the negative control si-NC/sh-NC or siRNAs/shRNAs targeting DARS1-AS1. The protein level of E2F1, CCND1 and CCNE2 was determined by western blotting in the (F) U87 and (G) GSC272 cells, with/without RNAi-mediated DARS1-AS1 depletion. RT-qPCR analysis of the RNA level of the established regulators of cell cycle E2F1, CCND1 and CCNE2 in the (H) U87 and (I) GSC272 cells transfected/transduced with the negative control si-NC/sh-NC or siRNA/shRNA targeting YBX1. The protein level of E2F1, CCND1 and CCNE2 was determined by western blotting in the (J) U87 and (K) GSC272 cells, with/without RNAi-mediated YBX1 depletion. Data in **B**-**E** and **H**-**I** are shown as mean±SD (n=3). $^{**}P < 0.01$ by 1-way ANOVA with Dunnett's multiple-comparison test.

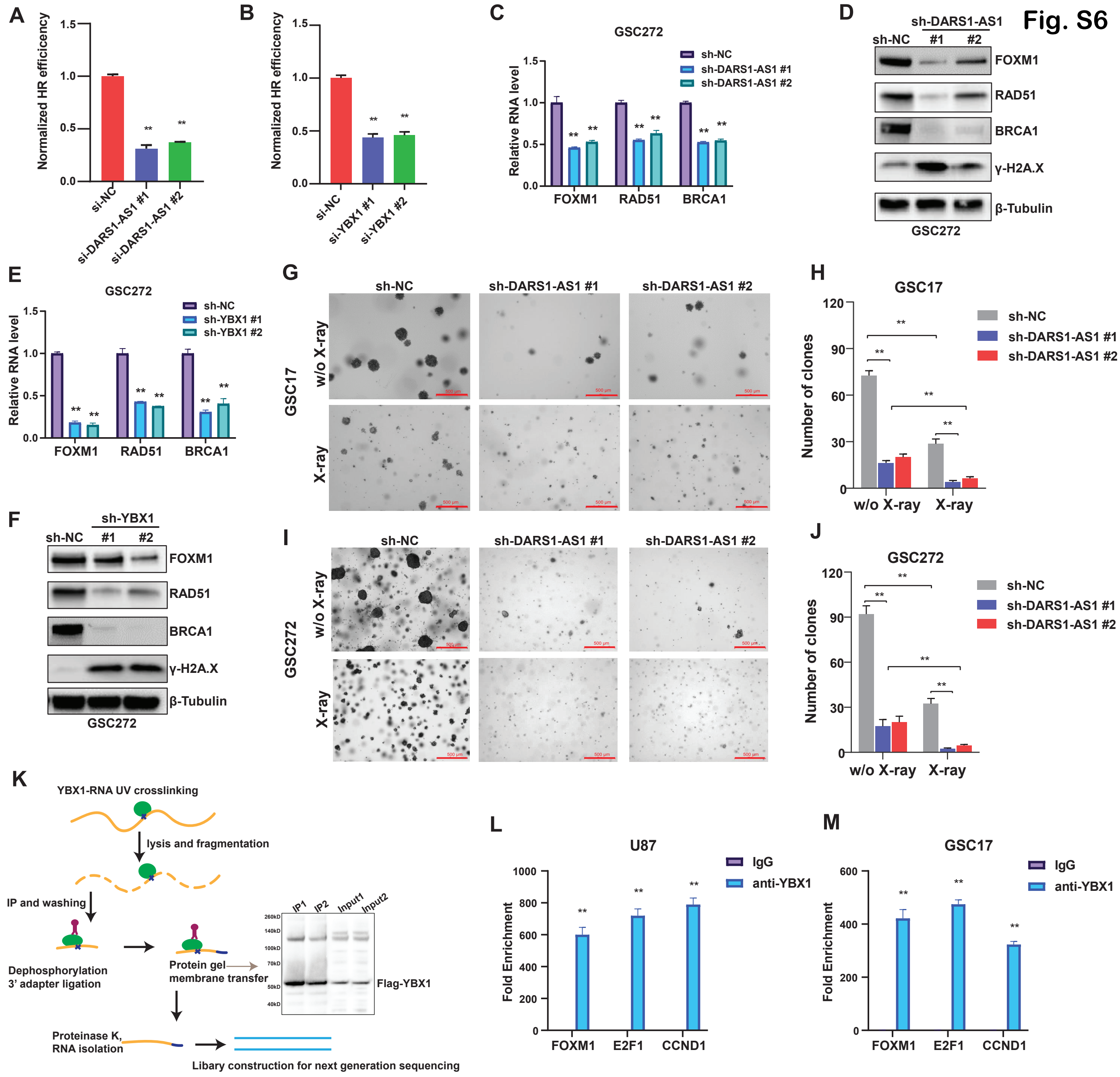
Fig. S6
A
Normalized HR efficiency
si-NC
si-DARS1-AS1 #1
si-DARS1-AS1 #2
B
Normalized HR efficiency
si-NC
si-YBX1 #1
si-YBX1 #2
C
GSC272
Relative RNA level
sh-NC
sh-DARS1-AS1 #1
sh-DARS1-AS1 #2
FOXM1
RAD51
BRCA1
D
sh-DARS1-AS1
sh-NC
#1
#2
FOXM1
RAD51
BRCA1
γ-H2A.X
β-Tubulin
GSC272
E
GSC272
Relative RNA level
sh-NC
sh-YBX1 #1
sh-YBX1 #2
FOXM1
RAD51
BRCA1
F
sh-YBX1
sh-NC
#1
#2
FOXM1
RAD51
BRCA1
γ-H2A.X
β-Tubulin
GSC272
G
sh-NC
sh-DARS1-AS1 #1
sh-DARS1-AS1 #2
GSC17
w/o X-ray
X-ray
H
GSC17
Number of clones
sh-NC
sh-DARS1-AS1 #1
sh-DARS1-AS1 #2
w/o X-ray
X-ray
I
sh-NC
sh-DARS1-AS1 #1
sh-DARS1-AS1 #2
GSC272
w/o X-ray
X-ray
J
GSC272
Number of clones
sh-NC
sh-DARS1-AS1 #1
sh-DARS1-AS1 #2
w/o X-ray
X-ray
K
YBX1-RNA UV crosslinking
lysis and fragmentation
IP and washing
Dephosphorylation
3' adapter ligation
Protein gel
membrane transfer
IP1
IP2
Input1
Input2
Flag-YBX1
Proteinase K,
RNA isolation
Libary construction for next generation sequencing
L
U87
Fold Enrichment
IgG
anti-YBX1
FOXM1
E2F1
CCND1
M
GSC17
Fold Enrichment
IgG
anti-YBX1
FOXM1
E2F1
CCND1

Figure S6. The effect of siRNA-mediated knockdown of (A) DARS1-AS1 or (B) YBX1 compared with the negative control siRNA (si-NC) on the homologous recombination (HR) repair efficiency was assessed using a DR-GFP reporter assay in U2OS cells (Materials and Methods). (C) RT-qPCR and (D) western blot analyses were performed to determine the RNA and protein level of the established regulator of homologous recombination-mediated DNA repair FOXM1, RAD51, and BRCA1 as well as the γ-H2AX level in the GSC272 cells transduced with sh-NC/DARS1- AS1 targeting shRNAs, respectively. (E) RT-qPCR and (F) western blot analyses were performed to determine the RNA and protein level of FOXM1, RAD51, and BRCA1 as well as the γ-H2AX level in the GSC272 cells transduced with sh-NC/YBX1 targeting shRNAs, respectively. (G) Representative pictures of soft agar colony formation and (H) bar graph quantifying the colonies formed in soft agar by the GSC17 cells that were transduced with the sh-NC or shRNAs targeting DARS1-AS1. (I) Representative pictures of soft agar colony formation and (J) bar graph quantifying the colonies formed in soft agar by the GSC272 cells that were transduced with the sh-NC or shRNAs targeting DARS1- AS1. (K) Schematic diagram of the YBX1 eCLIP-seq experiments and western blot showing the enrichment of the FLAG tagged YBX1 in the UV-crosslinked FLAG-tagged YBX1-RNA complex that was immunoprecipitated from the U251 cells in duplicate (IP1 and IP2). The whole cell lysate of the UV-crosslinked U251 cells in duplicate was used as input controls (Input1 and Input2). RNA immunoprecipitation (RIP) with an anti-YBX1/anti-IgG antibody followed by RT-qPCR validated the association of YBX1 with E2F1, CCND1 and FOXM1 mRNA in (L) U87 and (M) GSC17 cells, where anti-IgG antibody was used as a negative control. Data in **A-C** and **E** are shown as mean± SD (n=3). $^{**}P < 0.01$ by 1-way ANOVA with Dunnett's multiple-comparison test. Data in **H**, **J**, and **L-M** are shown as mean±SD (n=3). $^{**}P < 0.01$ by Student's *t*-test.

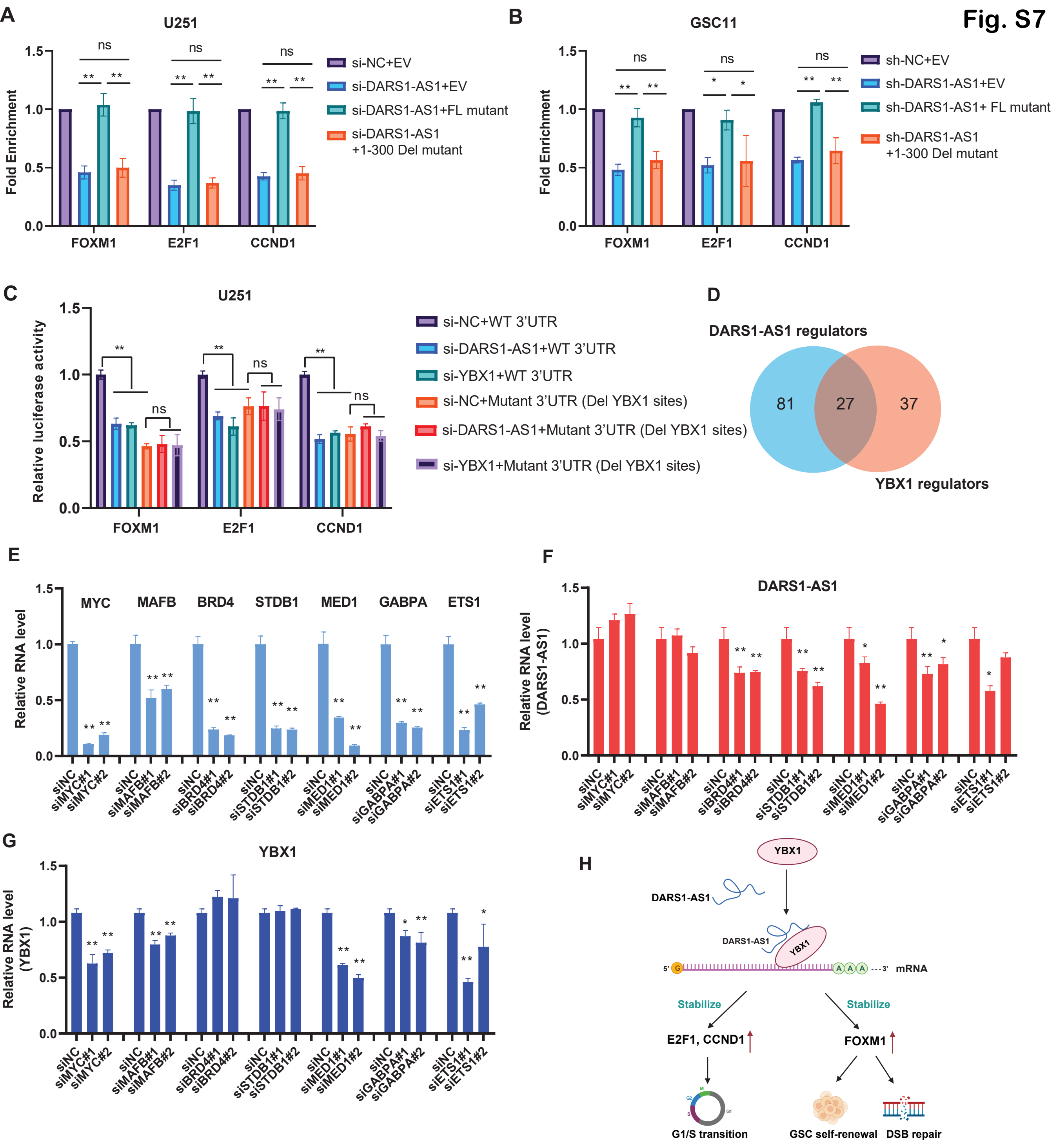

Fig. S7
A
U251
Fold Enrichment
FOXM1
E2F1
CCND1
si-NC+EV
si-DARS1-AS1+EV
si-DARS1-AS1+FL mutant
si-DARS1-AS1 +1-300 Del mutant
B
GSC11
sh-NC+EV
sh-DARS1-AS1+EV
sh-DARS1-AS1+ FL mutant
sh-DARS1-AS1 +1-300 Del mutant
C
U251
Relative luciferase activity
si-NC+WT 3'UTR
si-DARS1-AS1+WT 3'UTR
si-YBX1+WT 3'UTR
si-NC+Mutant 3'UTR (Del YBX1 sites)
si-DARS1-AS1+Mutant 3'UTR (Del YBX1 sites)
si-YBX1+Mutant 3'UTR (Del YBX1 sites)
D
DARS1-AS1 regulators
81
27
37
YBX1 regulators
E
Relative RNA level
MYC
MAFB
BRD4
STDB1
MED1
GABPA
ETS1
F
DARS1-AS1
Relative RNA level (DARS1-AS1)
G
YBX1
Relative RNA level (YBX1)
H
YBX1
DARS1-AS1
mRNA
Stabilize
E2F1, CCND1
FOXM1
G1/S transition
GSC self-renewal
DSB repair

Figure S7. The fold enrichment of the YBX1 RIP signal normalized by the input on E2F1, CCND1 and FOXM1 mRNA was determined in the (A) U251 and (B) GSC11 cells that transfected with si-NC/sh-NC and empty vector (EV) or siRNA/shRNA targeting DARS1-AS1 with siRNA/shRNA resistant full length mutant /1-300 deleted mutant of DARS1-AS1. (C) Luciferase reporter assays demonstrating the binding of YBX1/DARS1-AS1 to 3'UTR of targeted mRNAs. The wild type of 3'UTR of FOXM1, E2F1, CCND1 and their YBX1 binding sites deleted mutants were cloned into downstream of promoter and luciferase transcription unit. The indicated reporter plasmid and siRNA were co-transfected into U251 cells and luciferase (firefly/ Renilla) activity were measured. (D) Venn diagram showing the overlap between predicated regulators that regulate the expression of DARS1-AS1 and YBX1. (E) RT-qPCR analysis the knockdown efficiency of siRNAs targeting MYC, MAFB, BRD, STDB1, MED, GABPA and ETS1 in U251 cells. The RNA level of DARS1-AS1 (F) and YBX1 (G) in U251 cells transfected with the negative control si-NC or siRNA targeting indicated regulators. (H) Schematic diagram illustrating the mechanism of YBX1/DARS1-AS1 regulating G1/S transition, GSC self-renewal and DSB repair. Data in **A**-**B** are shown as mean$\pm$SD (n=3). **$P < 0.01$ or ns, not significant ($P > 0.05$) by 1-way ANOVA with Turkey's multiple-comparison test. Data in **C**, and **E**-**G** are shown as mean$\pm$SD (n=3). **$P < 0.01$ or *$P < 0.05$ by 1-way ANOVA with Dunnett's multiple-comparison test.

Supplementary Table 1. The sgRNA information and CRISPRi screen results.

Supplementary Table 2. The information about the primers, sgRNAs, siRNAs, shRNAs, and antibody.

Supplementary Table 3. The list of proteins identified by RNA pull-down followed by MS.

Supplementary Table 4. The list of differentially expressed protein-coding genes identified by RNA-seq in GBM cells upon DARS1-AS1/YBX1 knockdown, the curated gene sets (C2) from the Molecular Signature Database that were identified by GSEA as significantly enriched ones from RNA-seq data upon DARS1-AS1 knockdown, and the genes shared between GSEA and those identified by RNA pull-down followed by MS.

Supplementary Table 5. The YBX1 eCLIP-seq peaks, annotation, top enriched motifs and associated genes.

Supplementary Table 6. The list of computationally identified regulators of DARS1-AS1, YBX1 and their potential common regulators ,using ChIP-seq datasets curated in Cistrome data browswer, as well as the ChIP-seq datasets that provide supporting evidence for the common regulators of DARS1-AS1 and YBX1.
